# PKM2 dephosphorylation by Cdc25A promotes the Warburg effect and tumorigenesis

**DOI:** 10.1038/ncomms12431

**Published:** 2016-08-03

**Authors:** Ji Liang, Ruixiu Cao, Yajuan Zhang, Yan Xia, Yanhua Zheng, Xinjian Li, Liwei Wang, Weiwei Yang, Zhimin Lu

**Affiliations:** 1 Key Laboratory of Systems Biology, CAS Center for Excellence in Molecular Cell Science, Institute of Biochemistry and Cell Biology, Shanghai Institutes for Biological Sciences, Chinese Academy of Sciences, Shanghai 200031, China; 2 Shanghai Key Laboratory of Molecular Andrology, Institute of Biochemistry and Cell Biology, Shanghai Institutes for Biological Sciences, Chinese Academy of Science, Shanghai 200031, China; 3 Brain Tumor Center and Department of Neuro-Oncology, The University of Texas MD Anderson Cancer Center, Houston, Texas 77030, USA; 4 The Institute of Cell Metabolism and Disease, Shanghai Key Laboratory of Pancreatic Cancer, Shanghai General Hospital, School of Medicine, Shanghai Jiaotong University, Shanghai 200080, China; 5 Department of Oncology, Renji Hospital, School of Medicine, Shanghai Jiaotong University, Shanghai 200127, China; 6 National Laboratory of Oncogene and Cancer Related Genes Foundation, Shanghai 200127, China; 7 Department of Molecular and Cellular Oncology, The University of Texas MD Anderson Cancer Center, Houston, Texas 77030, USA; 8 Cancer Biology Program, The University of Texas Graduate School of Biomedical Sciences at Houston, Houston, Texas 77030, USA

## Abstract

Many types of human tumour cells overexpress the dual-specificity phosphatase Cdc25A. Cdc25A dephosphorylates cyclin-dependent kinase and regulates the cell cycle, but other substrates of Cdc25A and their relevant cellular functions have yet to be identified. We demonstrate here that EGFR activation results in c-Src-mediated Cdc25A phosphorylation at Y59, which interacts with nuclear pyruvate kinase M2 (PKM2). Cdc25A dephosphorylates PKM2 at S37, and promotes PKM2-dependent β-catenin transactivation and c-Myc-upregulated expression of the glycolytic genes *GLUT1*, *PKM2* and *LDHA*, and of *CDC25A*; thus, Cdc25A upregulates itself in a positive feedback loop. Cdc25A-mediated PKM2 dephosphorylation promotes the Warburg effect, cell proliferation and brain tumorigenesis. In addition, we identify positive correlations among Cdc25A Y59 phosphorylation, Cdc25A and PKM2 in human glioblastoma specimens. Furthermore, levels of Cdc25A Y59 phosphorylation correlate with grades of glioma malignancy and prognosis. These findings reveal an instrumental function of Cdc25A in controlling cell metabolism, which is essential for EGFR-promoted tumorigenesis.

## Abstract (web summary)

Protein phosphatase Cdc25 controls cell cycle transitions by dephosphorylating CDK substrates. Here, the authors show that the Cdc25A isoform regulates glycolysis through dephosphorylation of pyruvate kinase PKM2, resulting in β-catenin activation and consequent upregulation of the transcription of glycolytic genes.

## Graphical abstract

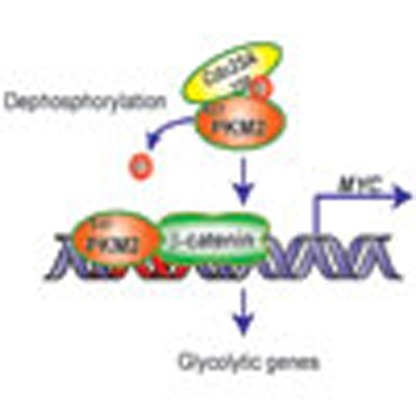

Protein kinases and phosphatases coordinate in the regulation of signalling responses to precisely mediate cellular activities^1^,^2^. Cell division cycle 25 (Cdc25), as a dual-specificity phosphatase, can remove phosphate groups from both phosphorylated tyrosine (Tyr, Y) and serine (Ser, S)/threonine (Thr, T) residues of its substrate proteins^3^. The only known substrate of Cdc25 is cyclin-dependent kinase (CDK)^4^. In mammalian cells, three isoforms of Cdc25 have been identified: Cdc25A, Cdc25B and Cdc25C^5^,^6^,^7^, which have been shown to control G1-S and G2-M transitions, and mitosis by spatially and temporally regulating their respective CDK substrates^4^,^8^. Cdc25A mainly promotes G1-S transition by dephosphorylating CDK4 (Y17)-cyclin D, CDK6 (Y24)-cyclin D and CDK2 (T14/Y15)-cyclin E/A complexes and G2-M progression by dephosphorylating CDK1 (T14/Y15)-cyclin A/B^9^,^10^,^11^,^12^. Cdc25B and Cdc25C primarily enable cell entry into mitosis by dephosphorylating CDK1 (T14/Y15)^13^,^14^,^15^. Cdc25A and Cdc25B have been reported to be overexpressed in various human cancers, including breast, ovarian, prostate, lung, colorectal, esophageal, thyroid, laryngeal, hepatocellular, gastric, pancreatic, endometrial, and head and neck cancers and non-Hodgkin lymphoma, neuroblastoma and glioma^4^. In addition, overexpression of Cdc25A and Cdc25B is often associated with high-grade tumours and poor prognosis^4^. The functions of Cdc25 in cell cycle control have been intensively studied^3^; however, whether Cdc25 has any non-CDK substrates that are involved in the regulation of other important cellular activities, such as cell metabolism, remains unclear.

Pyruvate kinase (PK) regulates the final step of glycolysis in the production of pyruvate and adenosine triphosphate (ATP)^16^,^17^. PKM1, PKM2, PKL and PKR are four pyruvate kinase isoforms that are expressed in different types of cells and tissues in mammals. Alternate splicing of *PKM* pre-mRNA by heterogeneous nuclear ribonucleoproteins (hnRNP) A1/2 and polypyrimidine-tract binding protein splicing factors leads to *PKM2* generation by the inclusion of exon 10 and the exclusion of exon 9, which is specific for *PKM1* (refs 18, 19). Besides its cytosolic roles in glycolysis^20^,^21^,^22^,^23^,^24^, PKM2 acts as a protein kinase and promotes cell cycle progression by regulating mitotic checkpoint, chromosome segregation and cytokinesis^25^,^26^. In addition, PKM2 upregulated by activation of growth factor receptor is phosphorylated at S37 by extracellular signal-regulated kinase (ERK)^27^,^28^. Phosphorylated PKM2 S37 recruits the peptidyl-proline isomerase protein interacting with never in mitosis A 1 (PIN1), resulting in the *cis–trans* isomerization and exposure of the nuclear localization signal of PKM2 and subsequent binding of importin α5 for nuclear translocation^28^.

In the nucleus, PKM2 binds to phosphorylated Y333 of β-catenin and activates β-catenin^16^,^29^. In addition, PKM2 is recruited to the promoter regions of β-catenin-regulated genes and phosphorylates histone H3 at T11. This phosphorylation results in H3-K9 acetylation and transcription of *CCND1* (encoding for cyclin D1), *MYC* and c-Myc-dependent GLUT1, lactate dehydrogenase A (LDHA), and polypyrimidine-tract binding that, in turn, promotes PKM2 expression^28^,^30^,^31^. The upregulated expression of the glycolytic genes enhances the Warburg effect while cyclin D1 expression promotes G1-S phase transition^29^,^30^. Thus, nuclear PKM2 regulates both cell metabolism and cell cycle progression. However, it is unclear whether nuclear PKM2 is post-translationally regulated for activation of gene transcription.

In this study, we found that nuclear PKM2 binds to c-Src phosphorylated Cdc25A at Y59, leading to Cdc25A-dependent PKM2 dephosphorylation, which is instrumental for PKM2 to interact with and activate β-catenin. β-catenin-mediated c-Myc expression subsequently upregulates expression of Cdc25A and glycolytic genes, which promotes the Warburg effect and cell proliferation.

## Results

### Nuclear PKM2 pS37 is dephosphorylated by Cdc25A

**Figure 1:**
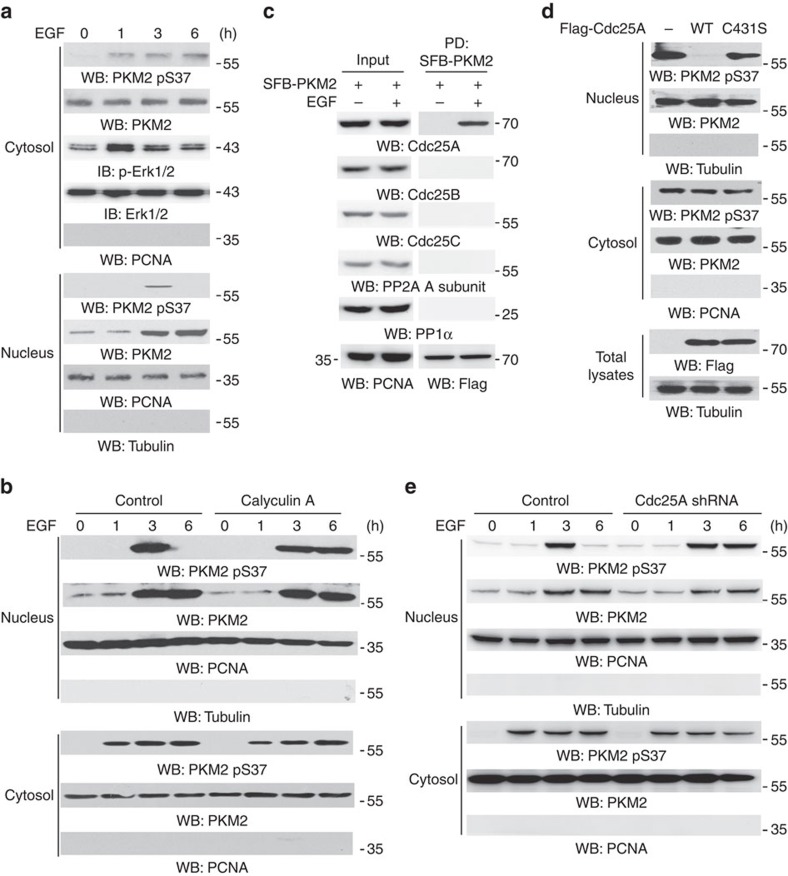
Nuclear PKM2 pS37 is dephosphorylated by Cdc25A. Immunoprecipitation and immunoblotting analyses were performed with the indicated antibodies. Data are representative of at least three independent experiments. (**a**) U87/EGFR cells were treated with or without EGF (100 ng ml^−1^) for the indicated period of time. Cytosolic and nuclear fractions of the cells were prepared. (**b**) U87/EGFR cells were pretreated with calyculin A (25 nM) for 30 min before EGF (100 ng ml^−1^) treatment for the indicated period of time. Cytosolic and nuclear fractions of the cells were prepared. (**c**) U87/EGFR cells stably expressing SFB-PKM2 were treated with or without EGF (100 ng ml^−1^) for 4 h. Nuclear lysates were prepared and followed by a pull-down assay of SFB-PKM2 with streptavidin-agarose beads. PD, pull-down. (**d**) U87/EGFR cells were infected with or without a lentivirus expressing Flag-Cdc25A WT or a catalytically inactive Cdc25A mutant (Cdc25A C431S) and were treated with EGF (100 ng ml^−1^) for 3 h. Cytosolic and nuclear fractions of the cells were extracted. (**e**) U87/EGFR cells expressing a control shRNA or shRNA against *Cdc25A* were treated with EGF (100 ng ml^−1^) for the indicated period of time. Cytosolic and nuclear fractions of the cells were extracted.

Epidermal growth factor receptor (EGFR) activation induces ERK-mediated PKM2 S37 phosphorylation in the cytosol, which results in nuclear translocation of about 10% cytosolic PKM2 (ref. 28). To examine whether PKM2 phosphorylation is dynamically regulated in the nucleus, we performed cell fraction analyses, which showed that EGF treatment of EGFR-overexpressing U87 (U87/EGFR) (Fig. 1a) or U251 (Supplementary Fig. 1a) human glioblastoma (GBM) cells for 3 h resulted in the nuclear translocation of PKM2 with S37 phosphorylation. However, phosphorylation levels were lower after prolonged EGF treatment, with no reduction in the total amount of PKM2 in the nucleus. In contrast, EGF treatment-induced PKM2 S37 phosphorylation in the cytosol, which corresponded to ERK activation, was detected at 1 h after treatment and remained at a higher level with prolonged EGF stimulation. Treatment with calyculin A (Fig. 1b) phosphatase inhibitor blocked PKM2 pS37 dephosphorylation in the nucleus upon EGF treatment for 6 h, suggesting the involvement of phosphatase activity in the regulation of nuclear PKM2 S37 phosphorylation.

To identify the involved phosphatase, we used streptavidin-agarose beads to pull-down nuclear S-FLAG-streptavidin-binding peptide (SFB)-tagged PKM2 and performed immunoblotting analyses with antibodies against nuclear protein phosphatases that can dephosphorylate phosphorylated serine/threonine residues, including Cdc25A, Cdc25B, Cdc25C, PP2A and PP1 (ref. 32). Figure 1c shows that only Cdc25A was associated with PKM2. In addition, overexpression of Flag-tagged wild-type (WT) Cdc25A, but not that of a catalytically inactive Cdc25A C431S mutant, dephosphorylated PKM2 at pS37 upon EGF treatment for 3 h in U87/EGFR cells (Fig. 1d) and U251 cells (Supplementary Fig. 1b). In contrast, treatment with NSC95397, a Cdc25-specific phosphatase inhibitor (Supplementary Fig. 1c), or depletion of Cdc25A by expressing its shRNA (short hairpin RNA) in U87/EGFR (Fig. 1e) and U251 (Supplementary Fig. 1d) human GBM cells and GSC11 human primary GBM cells (Supplementary Fig. 1e) enhanced nuclear PKM2 S37 phosphorylation upon EGF treatment for 6 h. The specificity of Cdc25A shRNA was validated by the fact that the expression of shRNA-resistant Cdc25A in endogenous Cdc25A-depleted U87/EGFR cells restored the dephosphorylation of PKM2 pS37 (Supplementary Fig. 1f). These results indicate that Cdc25A dephosphorylates PKM2 pS37 in the nucleus.

We next analyzed the dynamic regulation of nuclear PKM2 pS37 dephosphorylation by Cdc25A. Serum-starved U87/EGFR cells exhibited PKM2 S37 phosphorylation in the nucleus after EGF treatment for 3 h, which promoted the entry of the cells into the G1 phase from the serum starvation-arrested G0 phase (Supplementary Fig. 1g). S37 phosphorylation of nuclear PKM2 was diminished after EGF treatment for 6 h, suggesting that dephosphorylation of PKM2 S37 in the nucleus is important for gene expression during the G1-S phases (Supplementary Fig. 1g,h). In contrast, CDK2 pY15 dephosphorylation by Cdc25A occurred after 1 h of EGF treatment and was sustained through 6 h of EGF treatment (Supplementary Fig. 1i). These results provide additional evidence that Cdc25A, which was activated before the dephosphorylation of nuclear PKM2, regulates nuclear PKM2 activity.

### PKM2 binds to c-Src-phosphorylated Cdc25A at Y59

**Figure 2:**
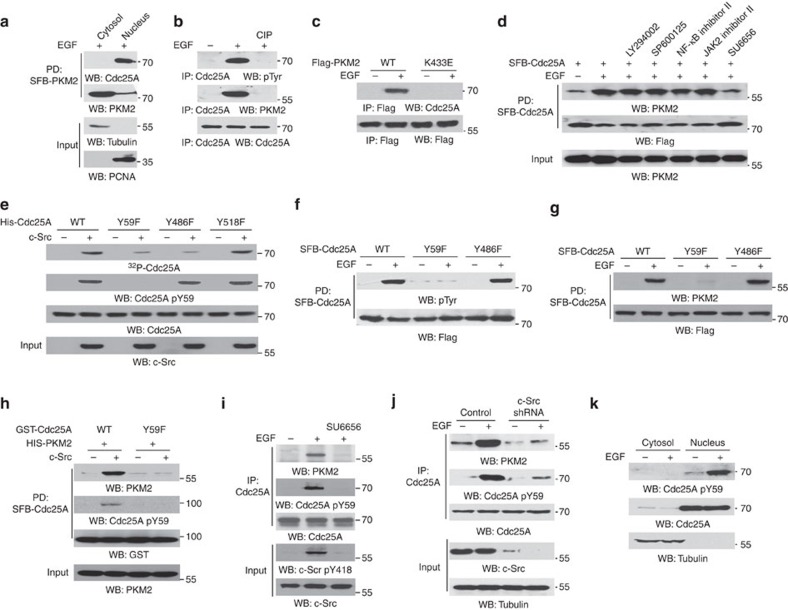
PKM2 binds to c-Src-phosphorylated Cdc25A at Y59. Immunoprecipitation and immunoblotting analyses were performed with the indicated antibodies. Data are representative of at least three independent experiments. (**a**) Cytosolic and nuclear factions were prepared from U87/EGFR cells stably expressing SFB-PKM2 after EGF (100 ng ml^−1^) treatment for 4 h. SFB-PKM2 was pulled down by streptavidin-agarose beads. (**b**) U87/EGFR cells were treated with or without EGF (100 ng ml^−1^) for 4 h. Immunoprecipitated Cdc25A in a 0.1% SDS lysis buffer (top panel) or a non-SDS lysis buffer (middle panel) was treated with or without CIP (10 unit) for 30 min at 37 °C followed by washing three times with PBS. (**c**) U87/EGFR cells transiently expressing WT Flag-PKM2 or Flag-PKM2 K433E were treated with or without EGF (100 ng ml^−1^) for 4 h. (**d**) U87/EGFR cells stably expressing SFB-Cdc25A were pretreated with LY294002 (20 μM), SP600125 (25 μM), NF-kB inhibitor II (7 μM), JAK2 inhibitor II (50 μM), SU6656 (4 μM) for 30 min, followed by EGF stimulation (100 ng ml^−1^) for 4 h. Pull-down of SFB-Cdc25A with streptavidin-agarose beads was performed. (**e**) An *in vitro* protein kinase assay was performed by mixing γ^32^P-ATP, purified active c-Src and indicated purified His-Cdc25A proteins. (**f**,**g**) U87/EGFR cells stably expressing SFB-Cdc25A WT, SFB-Cdc25A Y59F or SFB-Cdc25A Y486F were treated with or without EGF (100 ng ml^−1^) for 4 h. Pull-down of SFB-Cdc25A was performed with streptavidin-agarose beads. (**h**) An *in vitro* kinase assay was performed by mixing purified GST-Cdc25A WT or Y59F mutant with or without active c-Src. After the reaction, pulled down GST-Cdc25A was washed three times with PBS and incubated with purified recombinant His-PKM2 overnight. Pull-down of GST-Cdc25A with glutathione agarose beads was performed. (**i**) U87/EGFR cells were pretreated with SU6656 (4 μM) for 30 min before EGF (100 ng ml^−1^) treatment for 4 h. Immunoprecipitation of Cdc25A with anti-Cdc25A antibody was performed. (**j**) U87/EGFR with or without RNAi-mediated depletion of endogenous c-Src were treated with or without EGF (100 ng ml^−1^) for 4 h. Immunoprecipitation of Cdc25A with anti-Cdc25A antibody was performed. (**k**) Cytosolic and nuclear factions were prepared from U87/EGFR cells treated with or without EGF (100 ng ml^−1^) for 4 h.

Pull-down of SFB-tagged PKM2 from EGF-stimulated U87/EGFR cells showed that PKM2 interacted with Cdc25A in the nucleus, but not in the cytosol (Fig. 2a). In line with this finding, immunofluorescence analysis showed an increased colocalization between PKM2 and Cdc25A in the nucleus upon EGF treatment (Supplementary Fig. 2a). PKM2 binds to Tyr-phosphorylated proteins^29^,^33^. Immunoblotting of the immunoprecipitated Cdc25A showed that EGF stimulation resulted in Tyr phosphorylation of Cdc25A (Fig. 2b). Treatment of the immunoprecipitated Cdc25A with calf-intestinal alkaline phosphatase (CIP) dephosphorylated the Tyr-phosphorylated Cdc25A and abrogated the interaction between endogenous Cdc25A and PKM2. In addition, Flag-PKM2 K433E mutant, which has lost its ability to bind to Tyr-phosphorylated proteins^29^,^33^, was unable to bind to Cdc25A upon EGF stimulation (Fig. 2c). These results indicate that the binding of PKM2 to Cdc25A is dependent on Tyr phosphorylation of Cdc25A.

To identify the signal pathway or the protein kinase that regulates the interaction between Cdc25A and PKM2, we pretreated U87/EGFR cells with the following inhibitors: the phosphoinositide 3-kinase inhibitor LY294002, c-Jun N-terminal kinases (JNK) inhibitor SP600125, a NF-κB inhibitor, a JAK2 inhibitor and Src inhibitor SU6656, which blocked EGF-induced phosphorylation of AKT and c-Jun, TNF-α-induced and NF-κB-dependent IκBα promoter activation, and EGF-induced phosphorylation of Stat3 and c-Src, respectively (Supplementary Fig. 2b–f). Immunoblotting analyses showed that only inhibition of c-Src abrogated EGF-induced the binding of Cdc25A to PKM2 (Fig. 2d). Analysis of the Cdc25A amino acid sequence using PPSP (http://ppsp.biocuckoo.org) and DISPHOS (http://www.dabi.temple.edu) revealed that Y59, Y486 and Y518 are potential Tyr residues that can be phosphorylated by c-Src. An *in vitro* protein kinase assay by mixing γ^32^P-ATP, purified active c-Src and purified recombinant Cdc25A showed that c-Src phosphorylated Cdc25A (Fig. 2e). In contrast, c-Src-mediated phosphorylation was reduced in Cdc25A Y59F and Cdc25A Y486F, but not in Cdc25A Y518F mutant. In addition, combinational mutations of Y59 and Y486 into Phe abrogated c-Src-mediated phosphorylation of Cdc25A (Supplementary Fig. 2g), suggesting that Y59 and Y486 of Cdc25A are primary phosphorylation residues of c-Src *in vitro*.

To determine which residue of Cdc25A can be phosphorylated in cells and the role of phosphorylation of Y59 and Y486 of Cdc25A in binding of Cdc25A to PKM2, we expressed SFB-tagged Cdc25A WT, Cdc25A Y59F or Cdc25A Y486F in U87/EGFR cells and showed that Cdc25A Y59F, but not Cdc25A Y486F, largely blocked EGF-induced tyrosine phosphorylation of Cdc25A and the binding of Cdc25A to PKM2 (Fig. 2f,g). To further support the finding that Cdc25A Y59 phosphorylation is required for the interaction between PKM2 and Cdc25A, we performed an *in vitro* glutathione *S*-transferase (GST) pull-down assay by mixing purified His-PKM2 with purified recombinant WT GST-Cdc25A or GST-Cdc25A Y59F in the presence or absence of active c-Src. c-Src-mediated Cdc25A Y59 phosphorylation was detected by an anti-Cdc25A pY59 antibody (Fig. 2e,h), and the specificity of the antibody was validated by using a phosphorylation-specific blocking peptide (Supplementary Fig. 2h). As shown in Fig. 2h, PKM2 interacted with c-Src-phosphorylated Cdc25A WT and had limited association with non-phosphorylated Cdc25A WT or Cdc25A Y59F. In addition, treatment of U87/EGFR cells with EGF in different time points showed that the levels of Cdc25A Y59 phosphorylation was positively associated with amounts of Cdc25A-interacted PKM2 (Supplementary Fig. 2i). Furthermore, pretreatment with the Src inhibitor SU6656 (Fig. 2i) or depletion of endogenous c-Src (Fig. 2j) abrogated the EGF-induced interaction between PKM2 and Cdc25A, and Cdc25A Y59 phosphorylation. Notably, EGF induced Cdc25A Y59 phosphorylation in the nucleus but not in the cytosol (Fig. 2k). These results indicate that nuclear c-Src-dependent Cdc25A Y59 phosphorylation is required for the interaction between Cdc25A and PKM2.

### c-Src-phosphorylated-Cdc25A dephosphorylates nuclear PKM2

**Figure 3:**
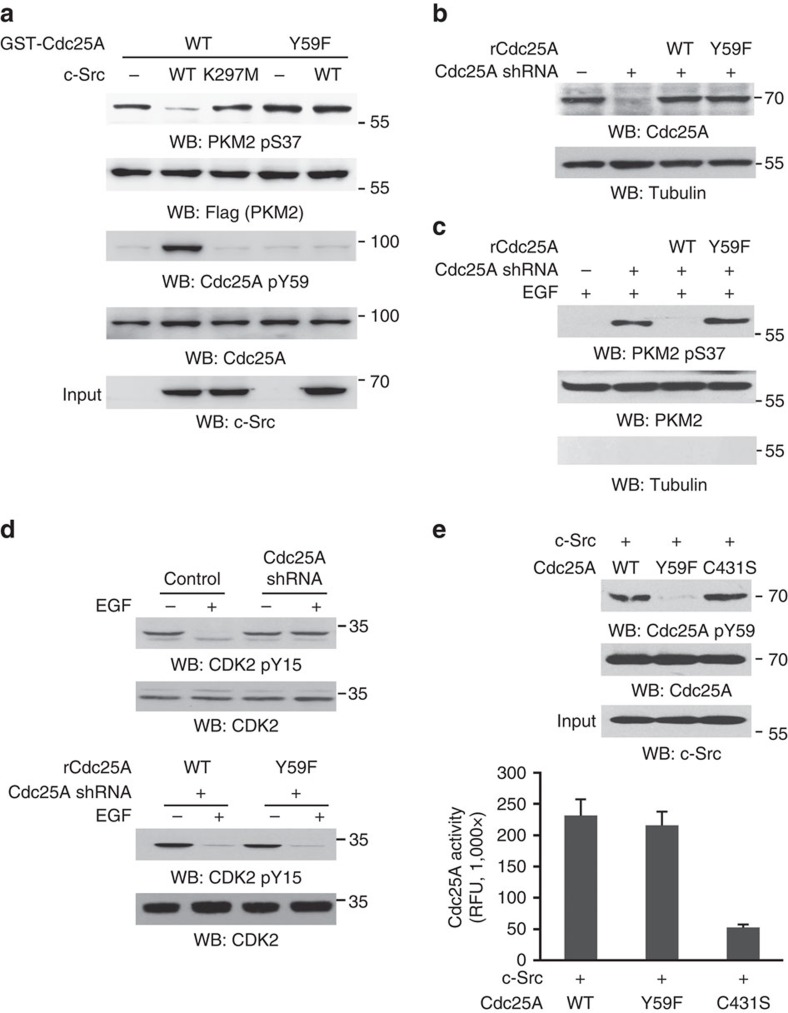
c-Src-phosphorylated Cdc25A Y59 dephosphorylates nuclear PKM2 pS37. Immunoprecipitation and immunoblotting analyses were performed with the indicated antibodies. Data are representative of at least three independent experiments. (**a**) Flag-PKM2, which was phosphorylated at Ser 37 by EGF treatment (100 ng ml^−1^) for 3 h in U87/EGFR, was immunoprecipitated and eluted by Flag peptides. Immobilized and purified WT GST-Cdc25A or GST-Cdc25A Y59F was mixed with or without wild-type c-Src or kinase-dead c-Src K297M for an *in vitro* kinase assay. After the reaction, GST-Cdc25A was pulled down and incubated with eluted Flag-PKM2 in a phosphatase buffer for dephosphorylation reaction. (**b**,**c**) Endogenous Cdc25A-depleted U87/EGFR cells were reconstituted with the expression of rCdc25A WT or rCdc25A Y59F (**b**). These cells were treated with EGF (100 ng ml^−1^) for 6 h. Nuclear fractions of the cells were extracted (**c**). (**d**) U87/EGFR cells with depleted Cdc25A and reconstituted the expression of rCdc25A WT or rCdc25A Y59F were treated with or without EGF (100 ng ml^−1^) for 30 min. (**e**) An *in vitro* kinase assay was performed by mixing Cdc25A WT, Y59F mutant or C431S mutant with active c-Src. After the reaction, immunoblotting analyses were performed with the reaction mixture (upper panel), or GST-Cdc25A WT and mutants were pulled down by glutathione agarose beads, and their activities toward FDP were examined using an Enzolyte FDP Protein Phosphatase Assay Kit (lower panel). Data represent the mean±s.d. of three independent experiments.

To determine the role of c-Src-dependent Cdc25A Y59 phosphorylation in PKM2 pS37 dephosphorylation, we performed an *in vitro* dephosphorylation assay. Figure 3a shows that both purified WT and Y59F mutant of Cdc25A failed to efficiently dephosphorylate immunoprecipitated and phosphorylated Flag-PKM2 pS37. However, the inclusion of purified active c-Src, but not c-Src K297M kinase-dead mutant, enabled Cdc25A WT, but not Cdc25A Y59F, to dephosphorylate PKM2 pS37 (Fig. 3a). These results were further confirmed by auto-radiography showing that ERK2-phosphorylated PKM2 was dephosphorylated by Cdc25A WT, but not Cdc25A Y59F, only in the presence of c-Src (Supplementary Fig. 3a). In addition, c-Src phosphorylated Cdc25A and accordingly affected PKM2 pS37 dephosphorylation in a dose-dependent manner (Supplementary Fig. 3b). These *in vitro* results were further supported by *in vivo* experiments. Reconstituted expression of RNAi-resistant (r) Cdc25A Y59F, but not its WT counterpart, in endogenous Cdc25A-depleted U87/EGFR (Fig. 3b), U251 and GSC11 cells (Supplementary Fig. 4a) enhanced PKM2 S37 phosphorylation in the nucleus in the presence of EGF treatment (Fig. 3c; Supplementary Fig. 4b). These results indicate that c-Src-dependent Cdc25A phosphorylation at Y59 is required for Cdc25A-dependent PKM2 pS37 dephosphorylation.

To examine the specificity of c-Src-dependent Cdc25A Y59 phosphorylation for dephosphorylation of PKM2 pS37, we examined the effect of Cdc25A expression on EGF-induced CDK2 Y15 phosphorylation. Figure 3d (upper panel) shows that EGF stimulation dephosphorylated CDK2 Y15 phosphorylation, and this dephosphorylation was blocked by Cdc25A depletion. However, reconstituted expression of rCdc25A WT and rCdc25A Y59F equally restored the dephosphorylation of CDK2 Y15 (lower panel), strongly suggesting that c-Src-dependent Cdc25A Y59 phosphorylation specifically regulates PKM2 pS37 dephosphorylation by Cdc25A. In line with these findings, an *in vitro* phosphatase activity assay showed that Cdc25A WT and Cdc25A Y59F compared with inactive Cdc25A C431S had comparable activity toward the dephosphorylation of phosphorylated peptides in the presence of active c-Src (Fig. 3e). In addition, c-Src did not affect Cdc25A to dephosphorylate CDK2 pY15 *in vitro* (Supplementary Fig. 4c). These results indicate that Cdc25A Y59 phosphorylation does not alter the enzymatic activity of Cdc25A or Cdc25A-depdendent CDK2 pY15 dephosphorylation.

### The binding of PKM2 to β-catenin requires PKM2 dephosphorylation

**Figure 4:**
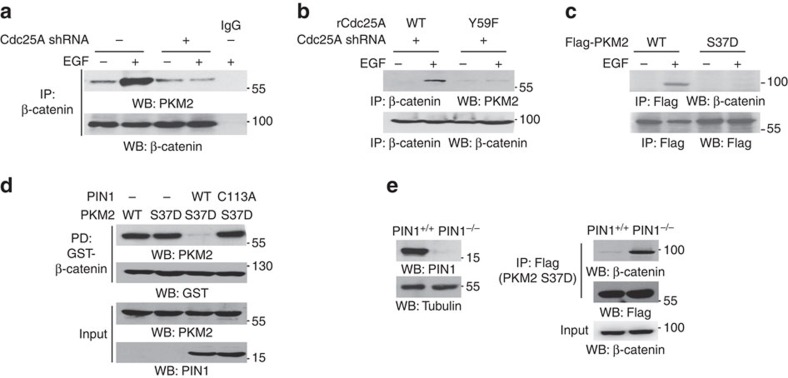
The binding of PKM2 to β-catenin requires PKM2 dephosphorylation. Immunoprecipitation and immunoblotting analyses were performed with the indicated antibodies. Data are representative of at least three independent experiments. (**a**,**b**) U87/EGFR cells with or without depleted Cdc25A (**a**) and reconstituted expression of rCdc25A WT or rCdc25A Y59F (**b**) were treated with or without EGF (100 ng ml^−1^) for 6 h. (**c**) U87/EGFR cells infected with a lentivirus expressing Flag-PKM2 WT or PKM2 S37D were treated with or without EGF (100 ng ml^−1^) for 6 h. (**d**) Purified recombinant His-PKM2 S37D was incubated with or without purified WT PIN1 or PIN1 C113A for *cis*–*trans* isomerization assay. Meanwhile, an *in vitro* kinase assay was performed by mixing immobilized GST-β-catenin and active c-Src. After the reactions, pulled down GST-β-catenin was washed with PBS three times and incubated with His-PKM2 S37D overnight at 4 °C, followed by a pull-down assay of GST-β-catenin with glutathione agarose beads. (**e**) PIN1 WT (PIN1^+/+^) or PIN1 knockout (PIN1^−/−^) cells were infected with lentivirus expressing Flag-PKM2 S37D. Immunoprecipitated Flag-PKM2 S37D from both cells was washed with PBS for three times and subsequently incubated with nuclear lysates of U87/EGFR cells treated with EGF for 4 h.

EGFR activation results in the binding of nuclear PKM2 to phosphorylated β-catenin and subsequent β-catenin transactivation^29^. To determine whether Cdc25A-mediated nuclear PKM2 dephosphorylation has any effect on the binding of PKM2 to β-catenin, we used EGF to treat U87/EGFR cells with or without Cdc25A depletion. As shown in Fig. 4a, Cdc25A depletion blocked the EGF-induced association between β-catenin and PKM2. In addition, reconstituted expression of rCdc25A Y59F mutant, unlike its WT counterpart, failed to rescue the binding of PKM2 to β-catenin (Fig. 4b). These results strongly suggest that PKM2 pS37 dephosphorylation is required for PKM2 to bind to β-catenin. These findings were further supported by co-immunoprecipitation analyses showing that the PKM2 S37D phosphorylation-mimic mutant, which is able to translocate into the nucleus^28^, failed to bind to β-catenin in the nucleus (Fig. 4c).

We previously showed that phosphorylated PKM2 S37 binds to PIN1, leading to PKM2 *cis–trans* conformational changes and exposure of the nuclear localization signal for importin α5 binding and nuclear translocation^28^. In the nucleus, PKM2 pS37 is dephosphorylated and is not regulated by PIN1 so that PKM2 is in the *cis* conformation, which may facilitate the binding of PKM2 to β-catenin. To examine whether PIN1-mediated structural change in PKM2 affects its binding to β-catenin, we conducted an *in vitro* isomerization assay by incubating purified His-PKM2 S37D with or without PIN1 WT or PIN1 C113A inactive mutant and an *in vitro* kinase assay by mixing purified GST-β-catenin and active c-Src. After the reactions, His-PKM2 S37D and GST-β-catenin were mixed together. Pull-down assay of GST-β-catenin with glutathione agarose beads showed that PKM2 S37D was able to interact with β-catenin (Fig. 4d). However, the presence of PIN1 WT, but not PIN1 C113A, blocked the association between PKM2 S37D and β-catenin. To further support this observation, we stably expressed Flag-PKM2 S37D in PIN1^+/+^ or PIN1^−/−^ cells. Immunoprecipitated Flag-PKM2 S37D from these cells was then incubated with nuclear lysates of EGF-treated U87/EGFR cells. Immunoblotting of the precipitated Flag-PKM2 S37D with anti-β-catenin antibody showed that Flag-PKM2 S37D from PIN1^+/+^ cells, but not from PIN1^−/−^ cells, failed to bind to β-catenin (Fig. 4e). These results indicate that PIN1-converted phosphorylated PKM2 in *trans* conformation is unable to bind to β-catenin and that PKM2 must be dephosphorylated in the nucleus to alleviate this effect.

### PKM2 dephosphorylation promotes β-catenin transactivation

**Figure 5:**
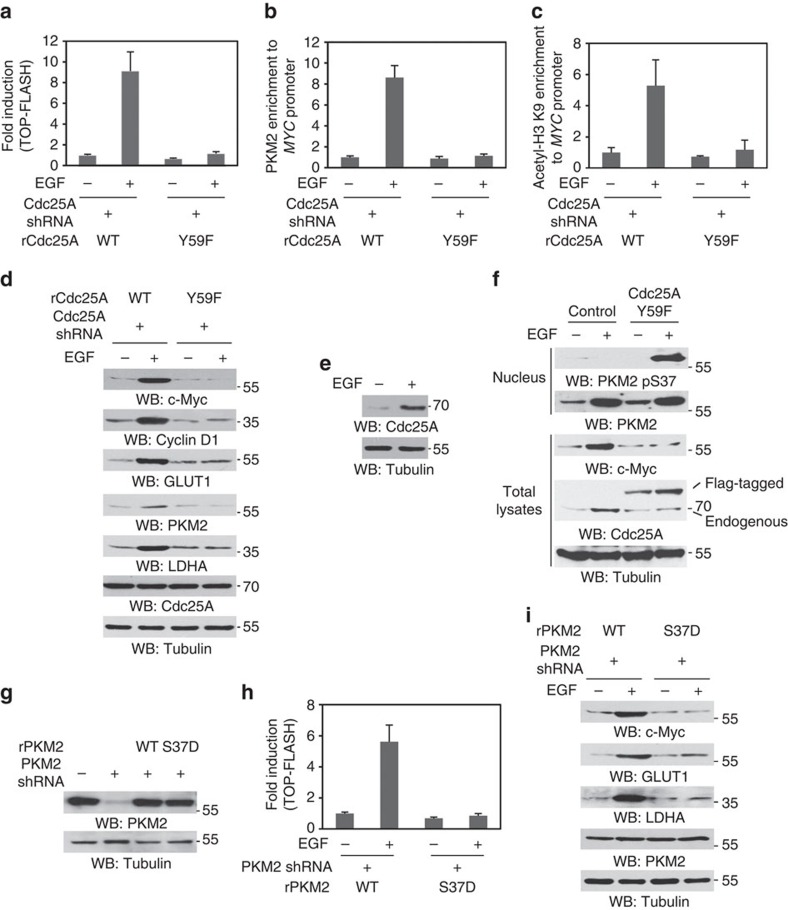
PKM2 dephosphorylation is required for β-catenin transactivation and c-Myc-dependent expression of glycolytic genes and Cdc25A. Immunoprecipitation and immunoblotting analyses were performed with the indicated antibodies. Data are representative of at least three independent experiments. (**a**) U87/EGFR cells with depleted Cdc25A and reconstituted expression of rCdc25A WT or rCdc25A Y59F were transfected with the TOP-FLASH plasmid and treated with or without EGF (100 ng ml^−1^) for 10 h. Data represent the mean±s.d. of three independent experiments. (**b**,**c**) U87/EGFR cells with depleted Cdc25A and reconstituted expression of rCdc25A WT or rCdc25A Y59F were infected with a lentivirus expressing Flag-PKM2 and then treated with or without EGF (100 ng ml^−1^) for 6 h. ChIP analyses with anti-Flag antibody (**b**) and anti-acetyl-H3-K9 antibody (**c**) and PCR with primers for *MYC* promoters were performed. Data represent the mean±s.d. of three independent experiments. (**d**) U87/EGFR cells with depleted Cdc25A and reconstituted expression of rCdc25A WT or rCdc25A Y59F were treated with or without EGF (100 ng ml^−1^) for 24 h. (**e**) U87/EGFR cells were treated with EGF (100 ng ml^−1^) for 24 h. (**f**) U87/EGFR cells were infected with or without a lentivirus expressing Flag-Cdc25A Y59F and treated with or without EGF (100 ng ml^−1^) for 6 h. Nuclear fractions were extracted for detection of nuclear PKM2 phosphorylation levels. Total lysates of the cells treated with EGF for 24 h were prepared for examination of c-Myc and Cdc25A expression. (**g**) Endogenous PKM2-depleted U87/EGFR cells were reconstituted with the expression of rPKM2 WT or rPKM2 S37D. (**h**) U87/EGFR cells with depleted PKM2 and reconstituted expression of rPKM2 WT or rPKM2 S37D mutant were transfected with the TOP-FLASH plasmid and treated with or without EGF (100 ng ml^−1^) for 10 h. Data represent the mean±s.d. of three independent experiments. (**i**) U87/EGFR cells with depleted PKM2 and reconstituted expression of rPKM2 WT or rPKM2 S37D were treated with or without EGF (100 ng ml^−1^) for 24 h.

To examine the effect of nuclear PKM2 pS37 dephosphorylation on β-catenin transactivation, we performed TCF/LEF-1 luciferase reporter analyses using the TOP-FLASH reporter plasmid containing multiple copies of TCF-binding sites for measuring β-catenin/transcription factor (TCF)/LEF-1 transcriptional activity in U87/EGFR cells with depleted Cdc25A and reconstituted expression of rCdc25A WT or rCdc25A Y59F (Fig. 3b). The expression of rCdc25A Y59F, but not its WT counterpart, significantly inhibited EGF-induced β-catenin transactivation (Fig. 5a). EGFR activation results in PKM2-dependent phosphosphorylation of histone H3-T11 leading to H3-K9 acetylation at *MYC* promoter regions^30^. Chromatin immunoprecipitation (ChIP) analyses showed that reconstituted expression of rCdc25A Y59F mutant but not its WT counterpart in endogenous Cdc25A-depleted U87/EGFR cells inhibited EGF-induced PKM2 recruitment to *MYC* promoter (Fig. 5b) and H3-K9 acetylation at *MYC* promoter regions (Fig. 5c). In line with this finding, reconstituted expression of rCdc25A Y59F mutant inhibited EGF-enhanced mRNA (Supplementary Fig. 5a) and protein expression (Fig. 5d) of *CCND1* and *MYC* and c-Myc-upregulated *GLUT1*, *PKM2* and *LDHA*. These results indicate that Cdc25A-dependent PKM2 dephosphorylation is instrumental for β-catenin transactivation and expression of cyclin D1 and c-Myc and downstream genes induced by c-Myc.

It is known that Cdc25A is transcriptionally regulated by c-Myc^34^. We next examined Cdc25A expression upon EGF treatment. As shown in Fig. 5e, EGF stimulation enhanced Cdc25A expression. To test whether Cdc25A can regulate itself through dephosphorylation of PKM2, we overexpressed Flag-Cdc25A Y59F in U87/EGFR cells. Overexpression of Flag-Cdc25A Y59F, which competitively blocked the interaction between c-Src and HA-Cdc25A WT and subsequent phosphorylation of HA-Cdc25A WT (Supplementary Fig. 5b), inhibited EGF-induced nuclear PKM2 pS37 dephosphorylation and upregulation of c-Myc, and endogenous Cdc25A expression (Fig. 5f). These results indicate that Cdc25A promotes its own expression in a feedback mechanism via dephosphorylation of PKM2 and upregulation of c-Myc.

The significance of PKM2 dephosphorylation in EGFR-induced gene expression was further supported by the finding that reconstituted expression of rPKM2 S37D, but not the expression of rPKM2 WT (Fig. 5g), blocked EGF-induced β-catenin transactivation measured by the TCF/LEF-1 luciferase reporter assay (Fig. 5h) and expression of c-Myc, GLUT1 and LDHA in endogenous PKM2-depleted U87/EGFR cells (Fig. 5i).

### PKM2 dephosphorylation promotes the Warburg effect

**Figure 6:**
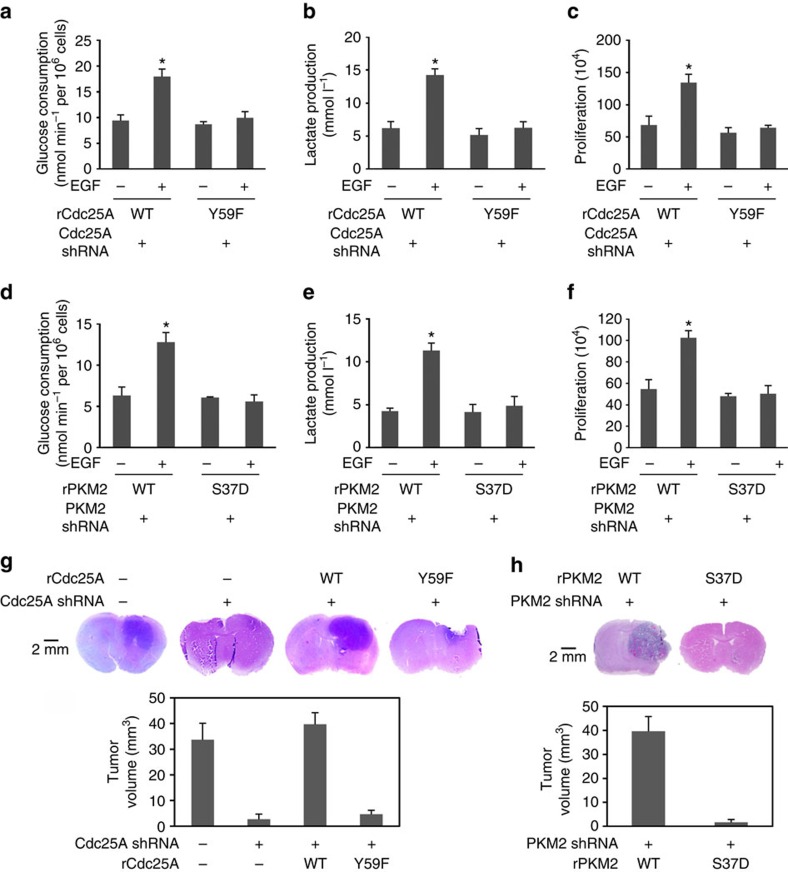
Cdc25A-mediated PKM2 pS37 dephosphorylation promotes the EGF-induced Warburg effect and tumorigenesis. (**a**,**b**,**d**,**e**) Cdc25A-depleted U87/EGFR cells with the reconstituted expression of rCdc25A WT or rCdc25A Y59F or PKM2-depleted U87/EGFR cells with the reconstituted expression of rPKM2 WT or rPKM2 S37D were incubated with no serum DMEM in the presence or absence of EGF (100 ng ml^−1^) for 20 h. The media were collected for analysis of glucose consumption (**a**,**d**) or lactate production (**b**,**e**), which was normalized by cell numbers (per 10^6^). Data represent the means±s.d. of three independent experiments. *P<0.05: statistically significant value in relation to the corresponding cells without EGF treatment. (**c**,**f**) A total number of 2 × 10^4^ Cdc25A-depleted U87/EGFR cells with the reconstituted expression of rCdc25A WT or rCdc25A Y59F or PKM2-depleted U87/EGFR cells with the reconstituted expression of rPKM2 WT or rPKM2 S37D were plated and counted 7 days after seeding in DMEM with 0.5% bovine calf serum in the presence or absence of EGF (100 ng ml^−1^). Data represent the means±s.d. of three independent experiments. *P<0.05: statistically significant value in relation to the corresponding cells without EGF treatment. (**g**,**h**) A total number of 5 × 10^5^ Cdc25A-depleted U87/EGFRvIII cells with the reconstituted expression of rCdc25A WT or rCdc25A Y59F (**g**), or PKM2-depleted U87/EGFRvIII cells with the reconstituted expression of rPKM2 WT or rPKM2 S37D (**h**) were intracranially injected into randomized athymic nude mice. After 2 weeks, the mice were euthanized and tumour growth was examined. Hematoxylin and eosin-stained coronal brain sections show representative tumour xenografts (top panel). Tumor volumes were calculated (bottom panel). Data represent the means±s.d. of seven mice.

EGFR activation promotes the Warburg effect^27^. Overexpression of Cdc25A in U87/EGFRvIII cells enhanced glucose consumption (Supplementary Fig. 6a), lactate production (Supplementary Fig. 6b) and cell proliferation (Supplementary Fig. 6c). In contrast, overexpression of Cdc25A Y59F (Supplementary Fig. 6d–f) or reconstituted expression of rCdc25A Y59F (Fig. 6a–c), rCdc25A C431S (Supplementary Fig. 6g–i) or rPKM2 S37D (Fig. 6d–f), but not their WT counterparts, in endogenous Cdc25A- or PKM2-depleted U87/EGFR cells largely inhibited EGF-induced enhancement of glucose consumption, lactate production and cell proliferation. In addition, reconstituted expression of rCdc25A Y59F in U87/EGFRvIII cells arrested the cells at G0/G1 phase (Supplementary Fig. 6j). In contrast to the dramatic effect of rPKM2 S37D expression on glycolysis and cell proliferation, purified PKM2 S37D had comparable glycolytic enzyme activity to its WT counterpart (Supplementary Fig. 7). These results strongly suggest that Cdc25A-mediated PKM2 pS37 dephosphorylation is required for the execution of the nuclear functions of PKM2 and the EGFR-promoted Warburg effect and cell proliferation.

To determine the role of Cdc25A-mediated PKM2 pS37 dephosphorylation in brain tumour development, we intracranially injected U87/EGFRvIII cells with or without depleted Cdc25A and reconstituted expression of rCdc25A or rCdc25A Y59F (Supplementary Fig. 6j) and U87/EGFRvIII cells with PKM2 depletion and reconstituted expression of rPKM2 WT or rPKM S37D (Supplementary Fig. 8a) into athymic nude mice. Dissection of the mouse brains 2 weeks after injection revealed that the animals injected with U87/EGFRvIII cells and U87/EGFRvIII cells with reconstituted expression of rCdc25A WT (Fig. 6g) or PKM2 WT (Fig. 6h) had rapid tumour growth. In contrast, no tumours or much smaller tumours were detected in the mice injected with U87/EGFRvIII cells with depleted Cdc25A or reconstituted expression of rCdc25A Y59F (Fig. 6g) or rPKM S37D mutant (Fig. 6h). Similar results were obtained by using GSC11 cells with depleted Cdc25A and reconstituted expression of rCdc25A WT or rCdc25A Y59F (Supplementary Fig. 8b). These results elucidate the significance of Cdc25A-mediated PKM2 pS37 dephosphorylation in EGFR-promoted brain tumour development.

### High Cdc25A pY59 levels indicate poor prognosis

**Figure 7:**
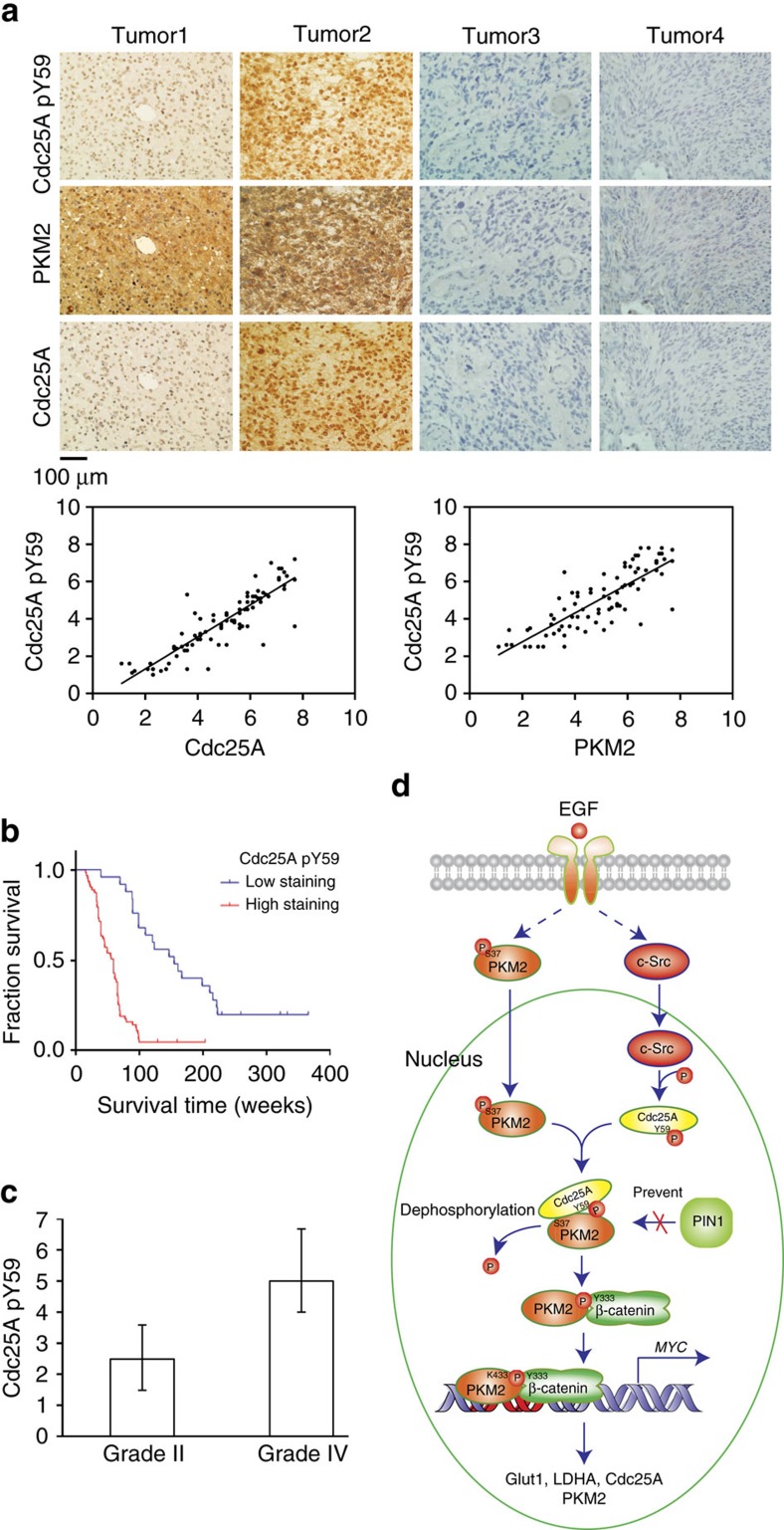
Cdc25A pY59 levels positively correlate with PKM2 and Cdc25A protein levels in human GBM samples and indicate prognosis. (**a**) Immunohistochemical staining of 88 GBM specimens with anti-phospho-Cdc25A-Y59, anti-PKM2 and anti-Cdc25A antibodies was performed. Top panel, representative photographs of four GBM specimens; bottom panel, semi-quantitative scoring (using a scale from 0 to 8) was carried out (Pearson product moment correlation test, left panel, *r*=0.88, *P*<0.001; right panel, *r*=0.82, *P*<0.001). (**b**) The survival times for 88 patients with low (0–4 staining scores, blue curve) versus high (4.1–8 staining scores, red curve) Cdc25A Y59 phosphorylation (low, 24 patients; high, 64 patients) were compared. Landmark represents censored (alive at last clinical follow-up) patients. (**c**) Thirty diffuse astrocytoma specimens were immunohistochemically stained with anti-phospho-Cdc25A-Y59 antibody, and specimens were compared with 88 stained GBM specimens (Student's *t* test, two-tailed, *P*<0.001). Data represent the mean±s.d. of 30 astrocytoma specimens and 88 GBM specimens. (**d**) A mechanism of Cdc25A-promoted Warburg effect and tumorigenesis. EGFR activation results in c-Src-dependent phosphorylation of Cdc25A at Y59. Phosphorylated Cdc25A binds to and dephosphorylates PKM2 pS37. PKM2 pS37 dephosphorylation is required for PKM2- and β-catenin-dependent c-Myc expression, which in turn promotes expression of glycolytic genes and Cdc25A, the Warburg effect and tumorigenesis.

To define the clinical relevance of our finding that Cdc25A regulates PKM2- and β-catenin-dependent gene expression, we performed immunohistochemistry (IHC) analyses of 88 human primary GBM specimens (World Health Organization (WHO) grade IV) with antibodies against Cdc25A pY59, PKM2 and Cdc25A. The antibody specificities were validated by using IHC analyses with specific blocking peptides or blocking recombinant proteins (Supplementary Fig. 9a) and immunoblotting analyses of the cell lysates of Cdc25A-depleted U87 cells (Supplementary Fig. 9b)^29^. Figure 7a showed that levels of Cdc25A pY59 correlated with the expression levels of PKM2 and Cdc25A, which are regulated by β-catenin-dependent c-Myc expression^28^. Quantification of the staining on a scale of 0–8 showed that these correlations were significant (Fig. 7a, bottom panel). In line with the results of c-Src-mediated Cdc25A phosphorylation and c-Myc-dependent PKM2 expression, IHC analyses of human primary GBM specimens revealed that the levels of c-Src pY418, Cdc25A pY59, c-Myc and PKM2 expression were correlated with each other (Supplementary Fig. 9c). Quantification of the staining on a scale of 0–8 showed that these correlations were significant (Supplementary Fig. 9d,e).

We next compared survival duration of 88 GBM patients, all of whom received surgery and standard adjuvant radiotherapy and temozolomide chemotherapy. Patients whose tumours had low (0–4 staining) Cdc25A Y59 phosphorylation (24 cases) had a median survival of 168 weeks; those whose tumours had high (4.1–8 staining) levels of Cdc25A Y59 phosphorylation (64 cases) had significantly lower median survival duration of 60 weeks (Fig. 7b). In addition, we examined whether the levels of Cdc25A Y59 phosphorylation correlated with the grades of glioma malignancy. We revealed that levels of Cdc25A Y59 phosphorylation in samples from patients (30 cases) with low-grade diffuse astrocytoma (WHO grade II; median survival time >5 years) were significantly lower than those from patients with high-grade GBM (Fig. 7c)^35^. These results support a role of Cdc25A Y59 phosphorylation in the clinical behaviour of human GBM and reveal a relationship between Cdc25A Y59 phosphorylation and clinical aggressiveness of glioma.

## Discussion

Cdc25A overexpression has been detected in many types of cancer and correlates with poor prognosis. However, to date, CDK is the only known substrates of Cdc25 for cell cycle regulation^3^,^4^. We show here that Cdc25A is involved in other cellular activities through dephosphorylating non-CDK signalling molecules. We demonstrate that Cdc25A is phosphorylated at Y59 by c-Src in response to EGFR activation. Phosphorylated Cdc25A Y59 binds to nuclear PKM2 and dephosphorylates PKM2 at phosphorylated S37, which is essential for the formation of the PKM2 and β-catenin complex, and for subsequent β-catenin transactivation, PKM2-dependent histone H3-T11 phosphorylation and H3-K9 acetylation, and c-Myc-upregulated expression of Cdc25A, GLUT1, PKM2 and LDHA. Cdc25A thereby regulates the EGFR-promoted Warburg effect, tumour cell proliferation and tumorigenesis (Fig. 7d). The finding that Cdc25A upregulates itself in a positive feedback mechanism via dephosphorylation of PKM2 and upregulation of c-Myc provides an instrumental insight into the role of overexpression of Cdc25A in cancer development.

The Cdc25 family has three isoforms in mammals: Cdc25A, Cdc25B and Cdc25C. Although Cdc25 is involved in cell cycle progression, each isoform has its specific function. Cdc25A mainly activates the CDK2-cyclin E and CDK2-cyclin A complexes during the G1-S transition, whereas Cdc25B and Cdc25C are primarily responsible for the activation of CDK1-cyclin A and CDK1-cyclin B at the G2-M transition^3^,^4^. Cdc25A and Cdc25B, but not Cdc25C, are overexpressed in human cancers^5^. In addition, *Cdc25b*^−/−^ female mice are sterile because Cdc25B is required for resumption of meiosis during oocyte maturation^36^. These findings indicate that each isoform of Cdc25 has some distinct cellular functions. In line with these observations, we found that c-Src phosphorylated the N-terminal regulatory domain of Cdc25A at Y59, and Y59 is not conserved in Cdc25B and Cdc25C. In addition, Cdc25A, but not Cdc25B and Cdc25C, interacts with and dephosphorylates nuclear PKM2 and subsequently regulates β-catenin-dependent gene expression. These results indicate that Cdc25A has a unique and instrumental role in regulating gene expression, which in turn regulates cell metabolism.

ERK1/2 phosphorylate PKM2 S37, leading to PIN1-dependent *cis–trans* isomerization of PKM2 and binding of importin α5 for nuclear translocation of monomeric PKM2. PKM2 S37D phosphorylation-mimic mutant is able to bind to PIN1 and subsequent importin α5 for nuclear translocation^28^,^37^. However, nuclear *cis*–*trans*-isomerized PKM2 S37D is unable to associate with β-catenin, suggesting that conformational change mediated by PIN1 inhibits monomeric PKM2 binding to β-catenin and that PKM2 pS37 has to be dephosphorylated in the nucleus to alleviate the effect of PIN1 on PKM2. These findings were further supported by *in vitro* biochemical studies showing that purified recombinant PKM2 S37D in the absence of, but not in the presence of, purified recombinant PIN1 and PKM2 S37D immunoprecipitated from PIN1^−/−^, but not from PIN1^+/+^, cells was able to interact with β-catenin. These results indicate that PKM2 phosphorylation status is precisely regulated and that Cdc25A plays an essential role in conformational regulation of PKM2 and PKM2-mediated β-catenin transactivation.

In the nucleus, PKM2 regulates gene expression by utilizing the phosphate group from (phosphoenolpyruvate) PEP but not ATP to phosphorylate histone H3 and STAT3 (refs 30, 38). PKM2 was also shown to promote mitosis and cytokinesis by phosphorylating Bub3 and myosin light chain 2, respectively^25^,^26^. In addition, more than 100 proteins were identified as substrates of PKM2 protein kinase activity, which can be enhanced by binding to succinyl-5-aminoimidazole-4-carboxamide-1-ribose-5′-phosphate (SAICAR)^39^. Although protein phosphorylation in PKM2-null lysates was not affected by recombinant PKM2, the protein kinase activity of PKM2 was demonstrated and validated by more recent publications. These reports show the yeast PKM2 homologue directly phosphorylates histone H3 at T11 and that PKM2-depdendent phosphorylation of mTORC1 inhibitor AKT1 substrate 1 (AKT1S1) results in mTORC1 activation^40^,^41^.

In summary, our findings reveal that PKM2 is a novel substrate of Cdc25A. We demonstrated that Cdc25A plays an instrumental role in the Warburg effect, in addition to its well-established role in cell cycle regulation. Given that Cdc25 has been targeted for cancer therapy, these findings may lead to an alternative approach involving intervention in Cdc25A function for the treatment of human cancers.

## Methods

### Materials

Rabbit polyclonal antibodies recognizing phospho-Cdc25A-Y59 (a customer antibody), phospho-PKM2 S37 (11456), PKM2 (21578), Cdc25C (21145) and c-Src (21168) were obtained from Signalway Antibody (College Park, MD, USA). Rabbit polyclonal antibodies recognizing Cdc25A (ab989) and CDK2 (ab7954) and rabbit monoclonal antibody of phospho-CDK2 Y15 (ab76146) were obtained from Abcam (Cambridge, MA, USA). Mouse antibodies recognizing phospho-tyrosine (05–321, clone 4G10) and phospho-c-Src Y418 (07–909) were obtained from Millipore (Billerica, MA, USA). Polyclonal antibodies for Cdc25B (sc-6948), β-catenin (sc-7963), GST (sc-138), and PCNA (sc-56) were purchased from Santa Cruz Biotechnology (Santa Cruz, CA, USA). EGF and mouse monoclonal antibodies for Flag (F3165), His (H1029), and tubulin (T9026) were purchased from Sigma (St Louis, MO, USA). A rabbit monoclonal antibodies against the PP2A A subunit (2041) and c-Myc (D3N8F) and a polyclonal antibody against PP1α (2582) were obtained from Cell Signaling Technology (Danvers, MA, USA). A polyclonal antibody specific for acetylated histone H3-K9 (07–352), hygromycin, puromycin, G418, DNase-free RNase A and propidium iodide were purchased from EMD Biosciences (San Diego, CA, USA). PolyJet *in vitro* DNA transfection reagent was from SignaGen Laboratories (Rockville, MD, USA). GelCode Blue Stain Reagent was obtained from Pierce (Rockford, IL, USA). Active c-Src was obtained from Signalchem (Richmond, BC, Canada).

The dilutions of the primary antibodies were: phospho-Cdc25A-Y59 (IHC: 1:400; WB: 1:1,000), phospho-PKM2 S37 (WB: 1:1,000), PKM2 (IF and IHC: 1:400; WB: 1:2,000), Cdc25C (WB: 1:1,000), c-Src (WB: 1:1,000), Cdc25A (IF and IHC: 1:400; WB: 1:1,000), CDK2 (WB: 1:1,000), phospho-CDK2 Y15 (WB: 1:1,000), phospho-CDK2 Y15 (WB: 1:500), phospho-tyrosine (WB: 1:2,000), phospho-c-Src Y418 (IHC: 1:400; WB: 1: 1,000), Cdc25B (WB: 1:1,000), β-catenin (WB: 1:2,000), GST (WB: 1:2,000), PCNA (WB: 1:2,000), Flag (WB: 1:5,000), His (WB: 1:2,000), tubulin (WB: 1:5,000), c-Myc (IHC: 1:400; WB: 1:1,000), PP1α (WB: 1:1,000), acetylated histone H3-K9 (WB: 1:1,000).

### Cell culture

Parental U87 and U251 cells were obtained from ATCC. Cell lines used in the experiments have been authenticated by short tandem repeat profiling. Mycoplasma contamination was examined using Cycleave PCR Mycoplasma Detection Kit (Takara). U87/EGFR, U87/EGFRvIII and U251 GBM cells, and 293T cells were maintained in Dulbecco's modified Eagle's medium (DMEM) supplemented with 10% bovine calf serum (HyClone, Logan, UT, USA). Human primary GSC11 GBM cells were maintained in DMEM/F-12 50/50 supplemented with B27, EGF (10 ng ml^−1^), and basic fibroblast growth factor (10 ng ml^−1^). Cell cultures were made quiescent by growing them to confluence, and the medium was replaced with fresh medium containing 0.5% serum for 24 h. EGF at a final concentration of 100 ng ml^−1^ was used for cell stimulation. The protein expression and reconstitution experiments were conducted using the established stable cell lines.

### DNA constructs and mutagenesis

Polymerase chain reaction (PCR)-amplified human PKM2 or Cdc25A were cloned into pcDNA3.1/hygro (+) vector between BamH I and Not I, pCDH-SFB vector between NheI and NotI, pColdI vector between BamHI and SalI, or pGEX-KG vector between BamHI and SalI. pcDNA3.1-Flag-PKM2 S37D, pcDNA3.1-Flag-PKM2 K433E, pCDH-SFB-Cdc25A Y59F, pCDH-SFB-Cdc25A Y486F, pCold I PKM2 S37D, pCold I Cdc25A Y59F, pCold I Cdc25A Y486F, pCold I Cdc25A C431S, pCold I Cdc25A Y59/481F, pCold I Cdc25A Y518F, pGEX-KG Cdc25A Y59F and pLOC-rCdc25A Y59F were made using the QuickChange Site-Directed Mutagenesis Kit (Stratagene, La Jolla, CA, USA). pLOC-rCdc25A contains non-sense mutations of A586G, T588C, C591T and T594C. pcDNA3.1-rPKM2 contains non-sense mutations of C1170T, C1173T, T1174C and G1176T.

The pGIPZ control was generated with the control oligonucleotide GCTTCTAACACCGGAGGTCTT. pGIPZ Cdc25A shRNA was generated with CAGGGAATTTCATTCCTCT. pGIPZ PKM2 shRNA was generated with CATCTACCACTTGCAATTA oligonucleotide targeting exon 10 of the *PKM2* transcript.

### Transfection

Overall, 4 × 10^5^ cells were cultured in 60-mm dish for 18 h and then transfected using Polyjet *In Vitro* DNA transfection Reagent (SignaGen) according to manufacturer's instructions.

### Subcellular fractionation analysis

U87/EGFR or U251 cells were collected and washed three times with cold phosphate buffered saline (PBS) buffer. Cells were resuspended gently in hypotonic buffer (20 mM Tris–HCl (pH 7.4), 10 mM NaCl and 3 mM MgCl_2_), incubated on ice for 15 min and then lysed by a Dounce homogenizer (40 strokes). The homogenate were centrifuged at 53*g* to remove intact cells and followed by a centrifugation at 800*g* to collect the nuclei. The supernatant contains the cytoplasmic fraction. The nuclei were washed three times in PBS and lysed by sonication.

### Cell proliferation assay

A total of 2 × 10^4^ cells were plated and counted 7 days after seeding in DMEM with 0.5% bovine calf serum. Data represent the mean±standard deviation of three independent experiments.

### Immunoprecipitation and immunoblotting analysis

Immunoprecipitation were performed with the lysates from indicated cultured cells and followed by the immunoblotting with corresponding antibodies. Briefly, the cells were collected and washed three times with cold PBS. Cell pellets were resuspended and lysed in the buffer (50 mM Tris–HCl (pH 7.4), 150 mM NaCl, 1% Triton X-100, 5 mM EDTA, 1 mM NaVO_3_, 50 mM NaF and protease inhibitor cocktail). The lysates were centrifuged at 15,000*g* to remove the cell debris. Supernatant were transferred a prechilled microcentrifuge tube. Protein concentration was determined using the BCA Protein Assay Kit (Pierce) according to the instruction of manufacturer. 1,500 μg of protein were incubated with indicated antibodies overnight and then mixed with protein A or protein G-agarose beads. Immunocomplexes were collected by centrifugation at 1,000*g* and resolved on SDS–PAGE gel and subsequently transferred to PVDF membranes (Millipore). The blots were blocked with 3% BSA followed by the incubation of primary antibodies and HRP-conjugated secondary antibodies. Blots were developed using SuperSignal West Pico and detected using Tanon 6600 Luminescent Imaging Workstation. Images have been cropped for presentation. Full-size images indicating the specificity of antibodies are presented in Supplementary Fig. 10.

### Purification of recombinant proteins

WT and mutant GST-Cdc25A, GST-β-catenin, His-Cdc25A, His-PKM2 and His-PIN1 were expressed and purified from bacteria. Briefly, BL21 cells transformed with indicated constructs were incubated at 37 °C (for pGEX or pET28 vectors) or 16 °C (for pCold I vectors) until the OD_600_ of the cultures reaches 0.6. 0.4 mM IPTG was added to induce the protein expression. Cell pellets were resuspended in PBS supplemented with protease inhibitor cocktail and lysed by sonication. Cell debris were removed by centrifugation at 15,000*g*. Supernatant was then incubated with glutathione agarose beads or Ni-charged agarose beads. Target proteins were recovered by centrifugation at 2,000*g*, eluted with imidazole (250 mM) or glutathione (20 mM) containing buffer, and dialyzed in PBS.

### *In vitro* kinase assay

The kinase reactions were performed as described previously^42^. Briefly, purified His-Cdc25A proteins (1 μg) were incubated with active c-Src (1 μg) (Signalchem, Canada) in the presence of 200 μM cold ATP in a kinase buffer (25 mM MOPS (pH 7.2), 12.5 mM β-glycerol-phosphate, 20 mM MgCl_2_, 25 mM MnCl_2_, 5 mM EGTA, 2 mM EDTA and 0.25 mM DTT) containing 10 μCi [γ-^32^P] ATP for 30 min at 30 °C. Reaction products were resolved by SDS–PAGE, and products labelled with ^32^P were visualized by auto-radiography.

### *In vitro* phosphatase assay

Immunoprecipitated Flag-PKM2 pS37 was eluted with Flag peptides and incubated with bacterial purified GST-Cdc25A (1 μg) proteins in the dephosphorylation reaction buffer (20 mM Tris–HCl (pH 8.5), 75 mM NaCl, 0.57 mM EDTA, 0.033% BSA and 1 mM DTT) for 1 h at 30 °C.

### 3,6-Fluorescein diphosphate (FDP) protein phosphatase assay

The phosphatase activity assays were performed according to the instruction of an Enzolyte FDP Protein Phosphatase Assay Kit (AnaSpec Co., Fremont, CA, USA). Briefly, GST-Cdc25A WT and mutants were pull-down by GST agarose beads, and their activity toward FDP was examined using the Enzolyte FDP Protein Phosphatase Assay Kit. The fluorescein signal was read using a fluorescence plate reader at excitation and emission wavelengths of 485 and 528 nm, respectively.

### Flow cytometry analysis

Overall, 1 × 10^6^ treated cells were fixed in cold 70% ethanol for 3 h, spun down, and incubated for 1 h at 37 °C in PBS with DNase-free RNase A (100 μg ml^−1^) and propidium iodide (50 μg ml^−1^). Cells were then analyzed by fluorescence-activated cell sorting.

### Luciferase reporter gene assay

The transcriptional activation of β-catenin in 293T cells was measured using Dual-luciferase Assay Kit (Promega) on GloMax 20/20 luminometer (Promega) following the manufacturer's instruction. The relative levels of luciferase activity were normalized to the levels of untreated cells and to the levels of luciferase activity of the Renilla control plasmid. Data represent the mean±s.d. of three independent experiments.

### Pyruvate kinase assay

The activity of bacterially purified PKM2 WT (0.1 μg) and PKM2 S37D (0.1 μg) toward PEP was measured with a Pyruvate Kinase Assay Kit (BioVision, Mountain View, CA, USA) according to the manufacturer's instruction. Data represent the mean±s.d. of three independent experiments.

### Quantitative real-time PCR

Total RNA was extracted with use of a RNeasy Plus Kit (Qiagen, Valencia, CA, USA). cDNA was prepared by using oligonucleotide (dT), random primers and a QuantiTect Reverse Transcription Kit (Qiagen). Quantitative real-time PCR analysis was performed under the following conditions: 5 min at 95 °C followed by 40 cycles at 95 °C for 30 s, 55 °C for 40 s, and 72 °C for 1 min using an ABI Prism 7700 sequence detection system. Data were normalized to expression of a control gene (β-actin) for each experiment.

The following primer pairs were used for quantitative real-time PCR: *PKM2*, 5′-GGGTTCGGAGGTTTGATG-3′ (forward) and 5′-ACGGCGGTGGCTTCTGT-3′ (reverse); *MYC*, 5′-ACACCCTTCTCCCTTCG-3′ (forward) and 5′-CCGCTCCACATACAGTCC-3′ (reverse); *CCND1*, 5′-GCGAGGAACAGAAGTGC-3′ (forward) and 5′-GAGTTGTCGGTGTAGATGC-3′ (reverse); *LDHA*, 5′-GGCCTGTGCCATCAGTATCT-3′ (forward) and 5′- GGAGATCCATCATCTCTCCC-3′ (reverse); *GLUT1*, 5′-ATGGAGCCCAGCAGCAA-3′ (forward) and 5′-ACTCCTCGATCACCTTCTGG-3′ (reverse); *β-actin*, 5′-ATGGATGACGATATCGCTGCGC-3′ (forward) and 5′-GCAGCACAGGGTGCTCCTCA-3′ (reverse).

### ChIP assay

ChIP assay was performed using SimpleChIP Enzymatic Chromatin IP Kit (Cell Signaling Technology) according to the instruction of manufacturer. Primer sequences used for the amplification of human *MYC* promoter were 5′-CAGCCCGAGACTGTTGC-3′ (forward) and 5′-CAGAGCGTGGGATGTTAG-3′ (reverse).

### Intracranial injection

We intracranially injected 5 × 10^5^ GBM cells (in 5 μl of DMEM per mouse) with endogenous Cdc25A depletion and reconstituted expression of rCdc25A WT or Y59F or GBM cells with endogenous PKM2 depletion and reconstituted expression of rPKM2 WT or S37D into randomized 4-week-old female athymic nude mice. The intracranial injections were performed. Seven mice per group in each experiment were included. Mice injected with U87/EGFRvIII or GSC11 cells were killed 14 or 30 days after glioma cell injection, respectively. The brain of each mouse was collected, fixed in 4% formaldehyde, and embedded in paraffin. Tumour formation and phenotype were determined by histologic analysis of hematoxylin and eosin-stained sections. The use of mice was in compliance with ethical regulations and was approved by the institutional review board at MD Anderson Cancer Center.

### Immunohistochemical analysis

Paraffin-embedded tumour tissues from human GBM and astrocytoma patients were stained with indicated antibodies. We scored tissue sections according to the percentage and intensity of staining, as previously defined^43^. We set the score of percentage as follows: 0, if no tumour sections were stained; 1, if<1% of sections were stained; 2, if 2–10% of sections were stained; 3, if 11–30% of sections were stained; 4, 31–70% of sections were stained; 5, 71–100% of sections were stained. The intensity of staining was rated on a scale of 0–3. The staining scores, representing the sum of percentage and intensity scores, were correlated with patient survival, defined as the time from date of diagnosis to death or last known date of follow-up. All patients received standard adjuvant radiotherapy after surgery, followed by the treatment of temozolomide. The uses of all patient samples in this study were approved by the University of Texas MD Anderson Cancer Center institutional review board. Informed consent was obtained from all patients. Data represent the mean±s.d. of 88 stained GBM specimens and 30 stained astrocytoma specimens.

### Statistical analysis

We determined the significance of differences in the human glioma data using the Pearson's correlation test and Student's *t* test (two-tailed). *P*<0.05 was considered to be significant.

### Data availability

Data supporting the findings of this study are available within the article and from the corresponding author upon reasonable request.

## Additional information

**How to cite this article:** Liang, J. *et al*. PKM2 dephosphorylation by Cdc25A promotes the Warburg effect and tumorigenesis. *Nat. Commun.* 7:12431 doi: 10.1038/ncomms12431 (2016).

## Supplementary Material

Supplementary Information Supplementary Figures 1-10
