# Supplementary material for: PKM2 dephosphorylation by Cdc25A promotes the Warburg effect and tumorigenesis
Source: Nat Commun. 2016 Aug 3;7:12431. doi: 10.1038/ncomms12431 (PMC4976202; doi:10.1038/ncomms12431)
Supplement: Supplementary Information — Supplementary Figures 1-10 [file ncomms12431-s1.pdf]

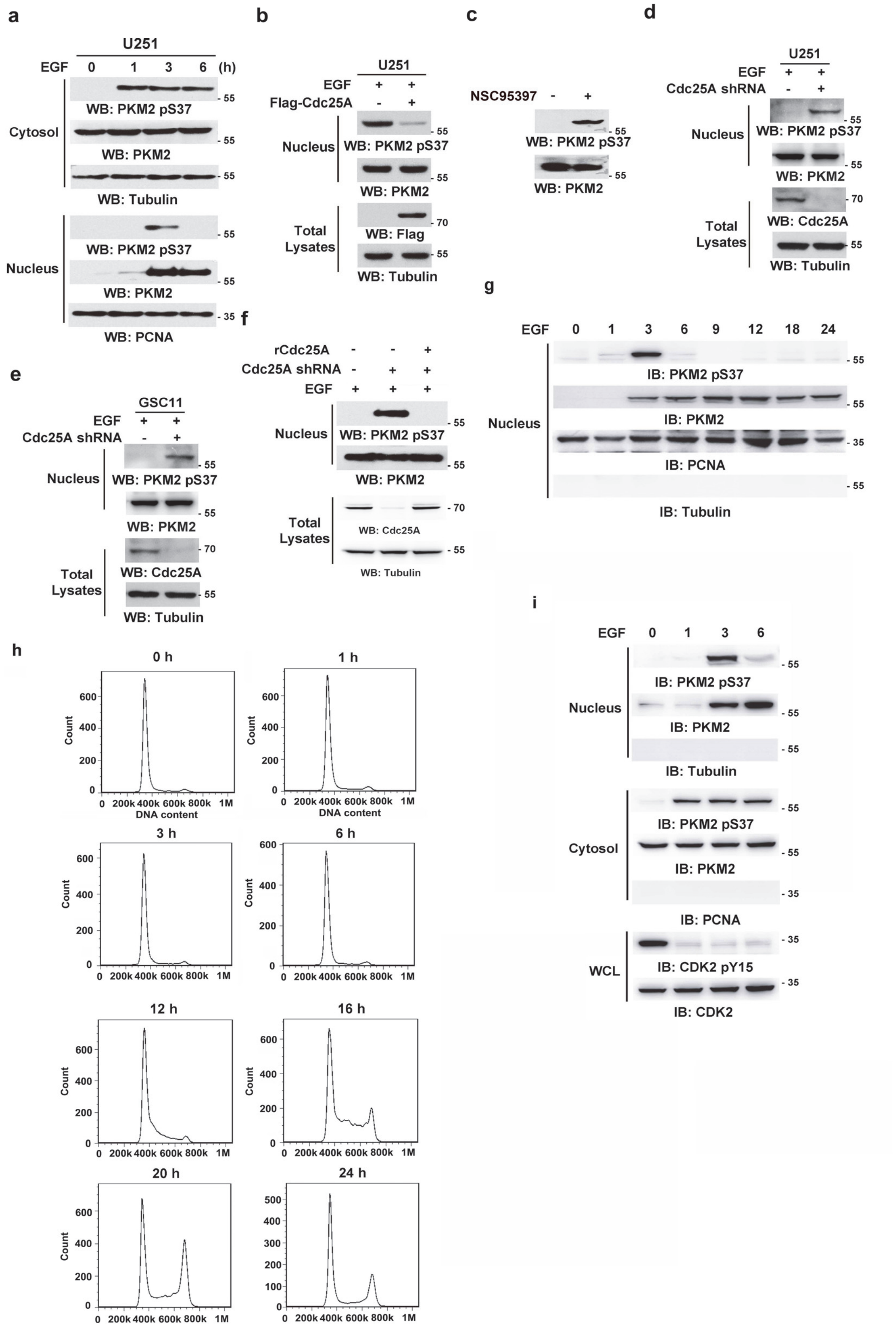

Supplementary Figure 1. Nuclear PKM2 pS37 is dephosphorylated by Cdc25A.

Immunoprecipitation and immunoblotting analyses were performed with the indicated antibodies. Data are representative of at least three independent experiments.

(**a**) U251 cells were treated with or without EGF (100 ng/ml) for the indicated period of time. Cytosolic and nuclear fractions of the cells were prepared.

(b) U251 cells with or without overexpressing Flag-Cdc25A were treated with EGF (100 ng/ml) for 3 h. Total cell lysates and nuclear lysates of the cells were prepared.

(c) U87/EGFR cells were pretreated with or without NSC95397 (25 μM) for 30 min before EGF (100 ng/ml) treatment for 6 h. Nuclear fractions of the cells were prepared.

(**d, e**) U251 (d) and GSC11 (e) cells expressing control shRNA or shRNA against *CDC25A* were treated without EGF (100 ng/ml) for 6 h. Nuclear fractions of the cells were extracted.

(**f**) U87/EGFR cells with depleted Cdc25A and reconstituted expression of rCdc25A WT were treated with EGF (100 ng/ml) for 6 h. Nuclear fractions of the cells were prepared.

(**g, h**) U87/EGFR cells were serum-starved for 24 h, and then treated with EGF (100 ng/ml) for indicated time. Nuclear fractions of the cells were prepared (g). Treated cells were harvested and analyzed for cell cycle distribution using flow cytometry (h).

(**i**) U87/EGFR cells were serum-starved for 24 h, and then treated with EGF (100 ng/ml) for indicated time. Cytosolic and nuclear fractions of the cells were prepared.

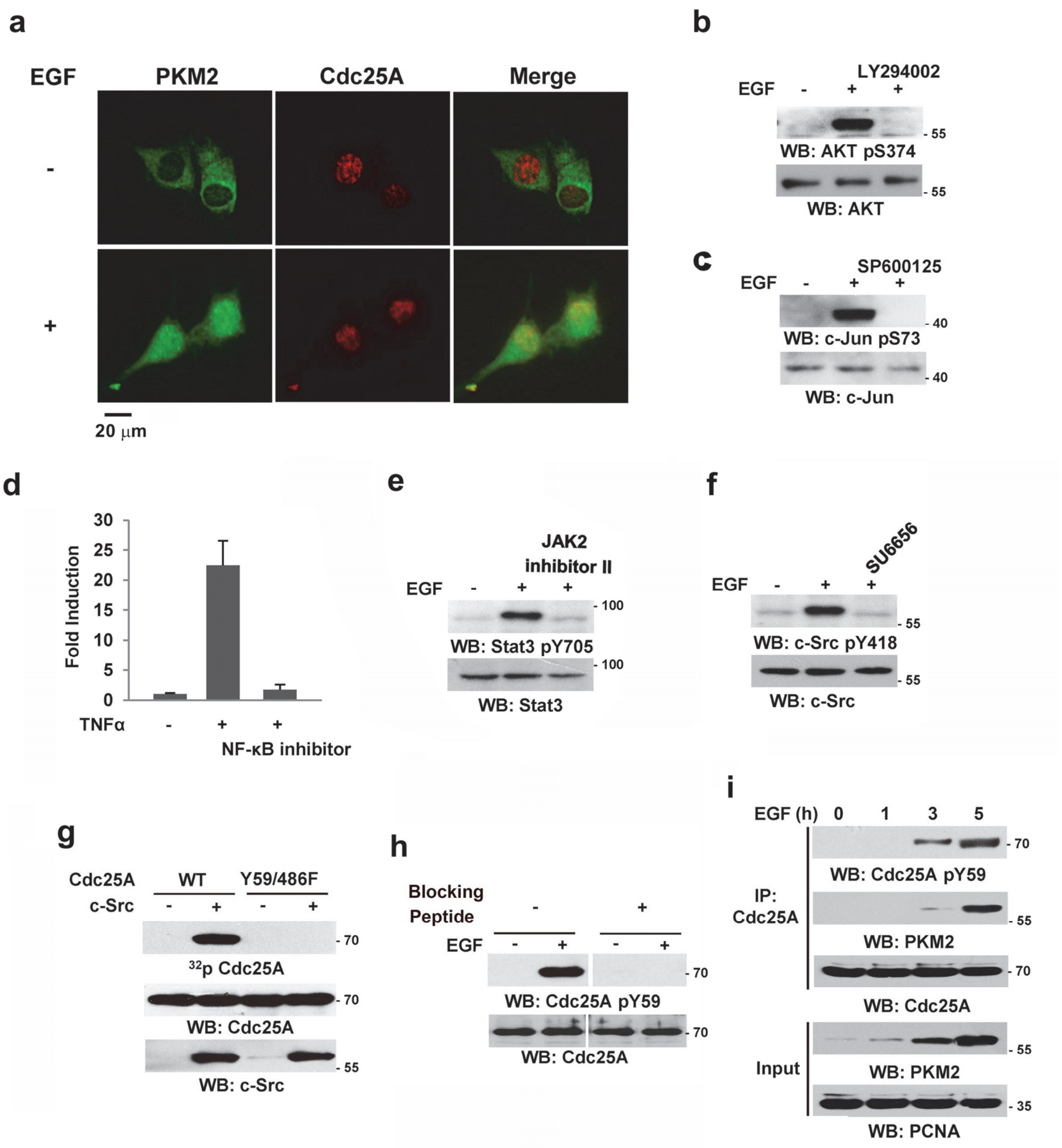

Supplementary Figure 2. PKM2 binds to c-Src-phosphorylated Cdc25A at Y59.

(**a**) U87/EGFR cells were treated with EGF (100 ng/ml) for 6 h. Immunofluorescence staining was performed by using the indicated antibodies.

(b, c, e, f) U87/EGFR cells were treated with LY294002 (20 μM) (b), SP600125 (25 μM) (c), JAK2 inhibitor II (50 μM) (e), SU6656 (4 μM) (f) for 30 min before EGF stimulation (100 ng/ml)

for 30 min. Immunoblotting analyses were performed with the indicated antibodies. Data are representative of at least three independent experiments.

(d) U87/EGFR cells transiently expressed a luciferase reporter vector containing the IκBα promoter. These cells were pretreated with or without NF-κB activation inhibitor II (7 μM) for 30 min before TNFα (10 ng/ml) treatment for 8 h. The relative levels of luciferase activity were normalized to the levels of untreated cells and to the levels of luciferase activity of the Renilla control plasmid. Data represent the mean ± standard deviation of three independent experiments.

(g) An in vitro protein kinase assay was performed by mixing γ^{32}P-ATP, purified active c-Src, and purified Cdc25A WT or Cdc25A Y59/486F mutant.

(**h**) U87/EGFR cells were treated with EGF (100 ng/ml) for 4 h. Immunoblotting analyses were performed with the indicated antibodies without (left panel) or with (right panel) a phosphorylation-blocking peptide.

(**i**) U87/EGFR cells were treated with EGF (100 ng/ml) for the indicated period of time. Nuclear fractions were prepared. Co-immunoprecipitation assay was performed with an anti-Cdc25A antibody.

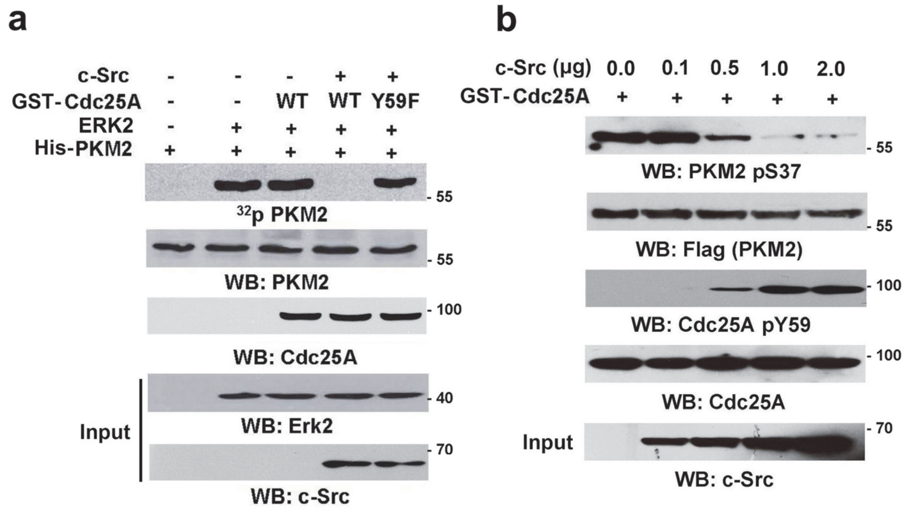

Supplementary Figure 3. c-Src-phosphorylated Cdc25A dephosphorylates nuclear PKM2 pS37.

Immunoblotting analyses were performed with the indicated antibodies. Data are representative of at least three independent experiments.

(**a**) In vitro kinase assay was performed by mixing γ^{32}P-ATP and purified His-PKM2 with or without active ERK2, or by mixing γ^{32}P-ATP and purified GST-Cdc25A WT or GST-Cdc25A Y59F with or without active c-Src. After the reaction, His-PKM2 and GST-Cdc25A pulled down from the reaction mixture were incubated together for an in vitro dephosphorylation assay.

(**b**) An in vitro kinase assay was performed by mixing purified GST-Cdc25A and indicated doses of active c-Src for 30 min. Flag-PKM2 was immunoprecipitated from EGF-treated (100 ng/ml, 3 h) U87/EGFR cells and eluted with Flag peptides. Then, PBS-washed and immobilized GST-Cdc25A was incubated with eluted Flag-PKM2 in a phosphatase buffer for an in vitro dephosphorylation assay.

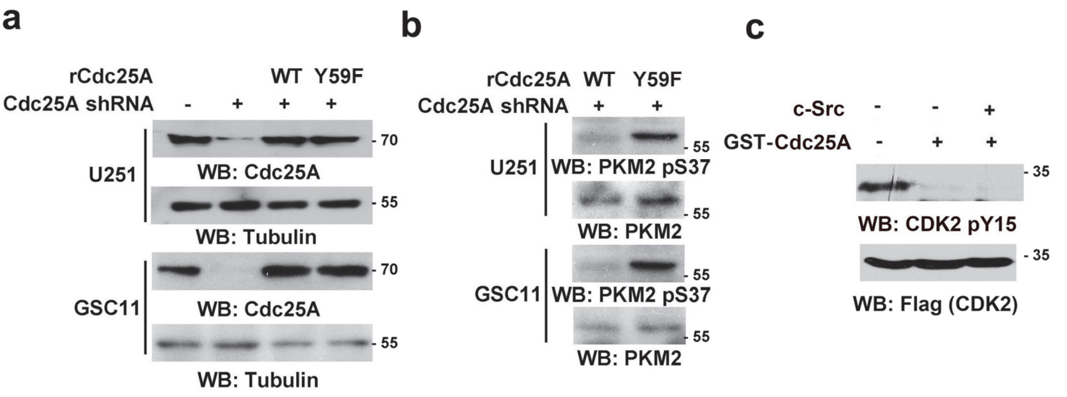

Supplementary Figure 4. Phosphorylation of Cdc25A at Y59 is required for the dephosphorylation of PKM2 in U251 and GSC11 cells.

(a, b) U251 and GSC11 cells with depleted endogenous Cdc25A were reconstituted with the expression of rCdc25A WT or rCdc25A Y59F (a). These cells were treated with EGF (100 ng/ml) for 6 h. Nuclear fractions of the cells were extracted (b).

(**c**) Purified GST-Cdc25A was incubated with or without active c-Src for an in vitro kinase assay. Flag-CDK2 was immunoprecipitated from EGF (100 ng/ml, 30 min)-treated U87/EGFR cells stably expressing Flag-CDK2 and eluted with Flag peptides. Then, GST-Cdc25A pulled down from the kinase reaction was incubated with eluted Flag-CDK2 for an in vitro dephosphorylation assay.

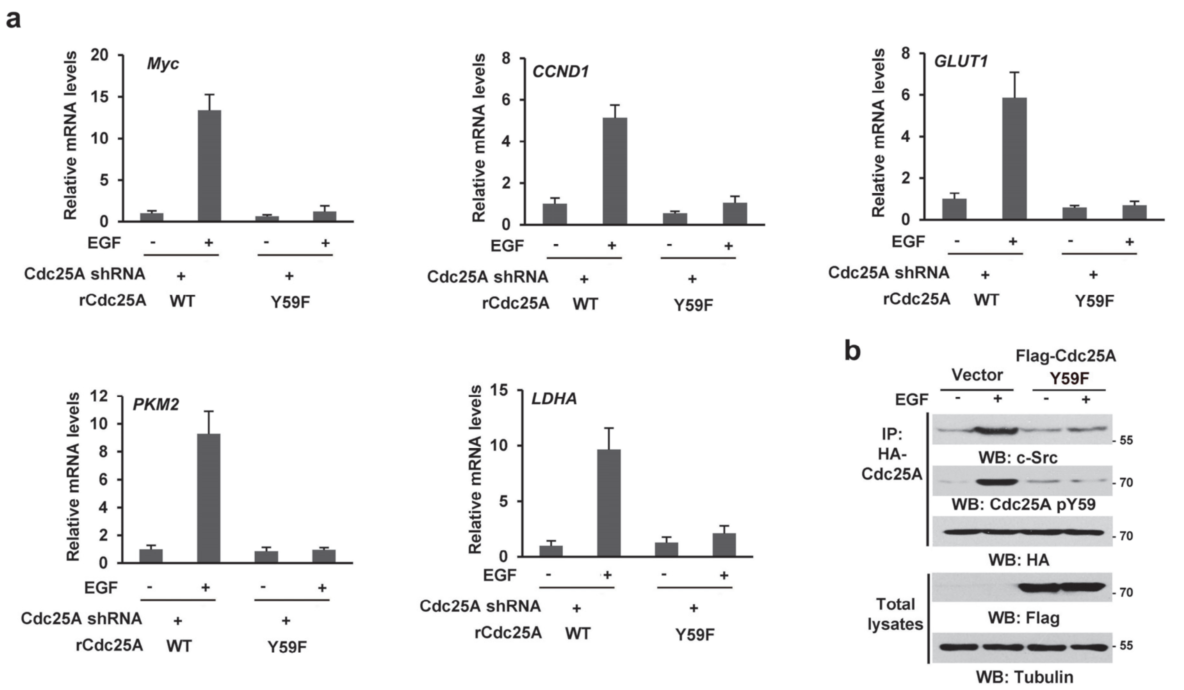

Supplementary Figure 5. PKM2 pS37 dephosphorylation is required for β-catenin transactivation and c-Myc-dependent expression of glycolytic genes.

(**a**) U87/EGFR cells with depleted Cdc25A and reconstituted expression of WT rCdc25A or rCdc25A Y59F were treated with or without EGF (100 ng/ml) for 24 h. Total mRNA was extracted and quantitative PCR was performed with indicated primer.

(b) U87/EGFR cells stably expressing HA-Cdc25A were infected with or without a lentivirus expressing Flag-Cdc25A Y59F. Co-immunoprecipitation was performed using the antibody of anti-HA. Immunoblotting analyses were performed with the indicated antibodies. Data are representative of at least three independent experiments.

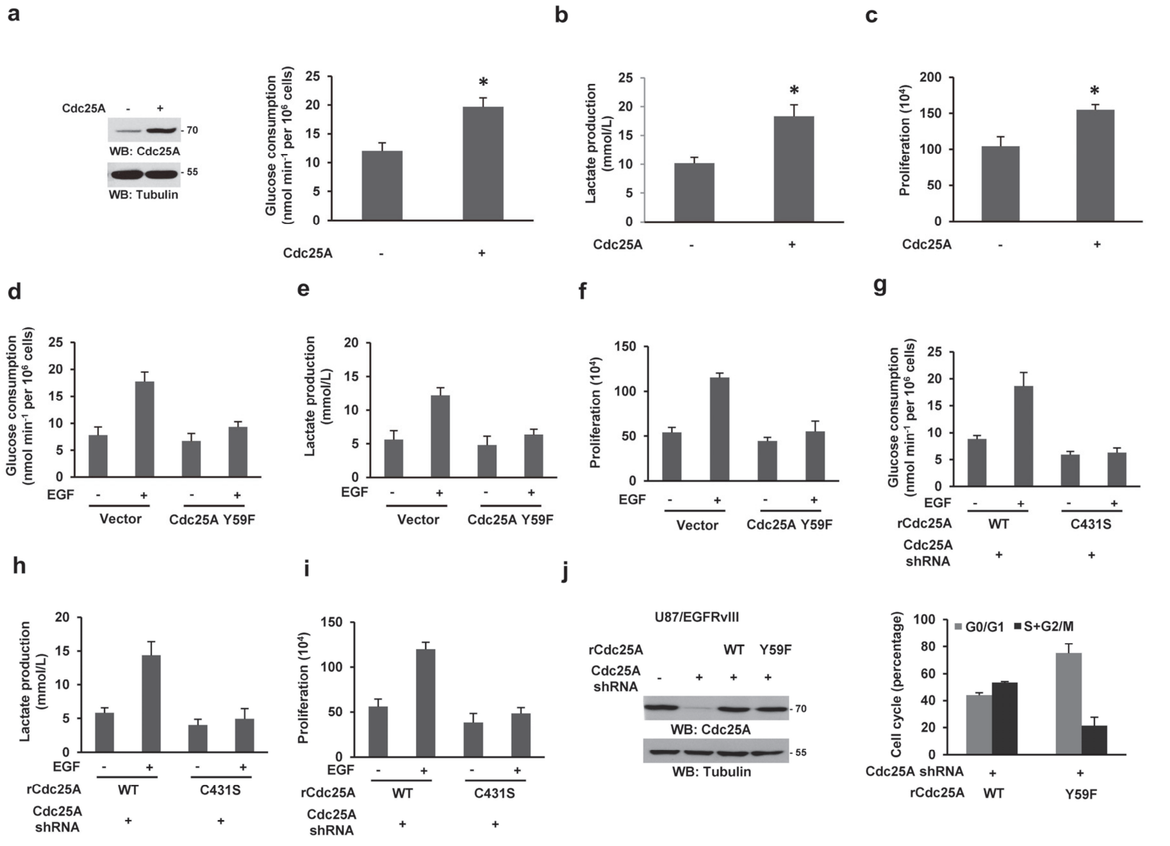

Supplementary Figure 6. Cdc25A expression enhances glucose consumption, lactate production, and cell proliferation.

(a-c) Cdc25A was overexpressed in U87/EGFRvIII cells. The glucose consumption (a), lactate production (b), and cell proliferation (c) were measured. Data represent the means ± SD of three independent experiments. Immunoblotting analyses were performed with the indicated antibodies.

(**d-f**) U87/EGFR cells with or without overexpression of Cdc25A Y59F were incubated with non-serum DMEM in the presence or absence of EGF (100 ng/ml) for 20 h. The glucose consumption (d), lactate production (e), and cell proliferation (f) were measured. Data represent the means ± SD of three independent experiments.

(**g-i**) Cdc25A-depleted U87/EGFR cells with reconstituted expression of rCdc25A WT or catalytically inactive mutant rCdc25A C431S were incubated with non-serum DMEM in the presence or absence of EGF (100 ng/ml) for 20 h. The glucose consumption (g), lactate production (h), and cell proliferation (i) were measured. Data represent the means ± SD of three independent experiments.

(**j**) U87/EGFRvIII cells with depleted endogenous Cdc25A were reconstituted with the expression of WT rCdc25A or rCdc25A Y59F. Cell cycle analysis was performed with the cells using flow cytometry. Immunoblotting analyses were performed with the indicated antibodies.

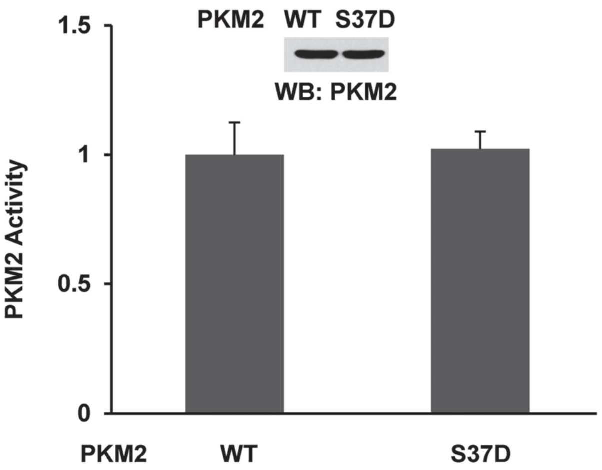

Supplementary Figure 7. WT PKM2 and PKM2 S37D have comparable glycolytic enzyme activity in vitro.

PKM2 glycolytic enzyme activity assay was performed with purified WT PKM2 and PKM2 S37D. Data represent the means ± SD of three independent experiments. Immunoblotting analyses were performed with the indicated antibodies.

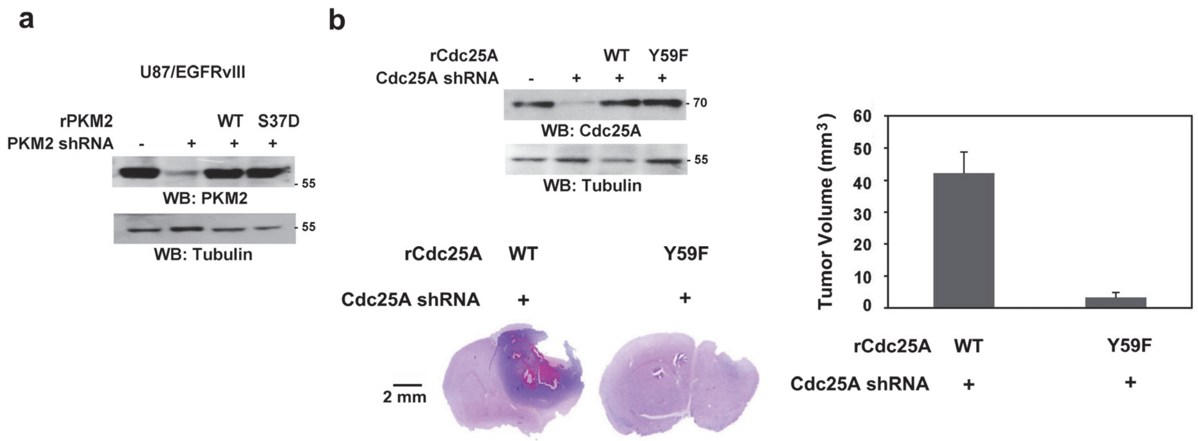

Supplementary Figure 8. Cdc25A-mediated PKM2 pS37 dephosphorylation promotes brain tumorigenesis.

(a) U87/EGFRvIII cells with depleted endogenous PKM2 were reconstituted with the expression of WT rPKM2 or rPKM2 S37D. Immunoblotting analyses were performed with the indicated antibodies.

(b) The indicated GSC11 cells (5×10^5) with depleted Cdc25A and reconstituted expression of WT rCdc25A or rCdc25A Y59F mutant (left upper panel) were intracranially injected into randomized athymic nude mice. After 4 weeks, the mice were sacrificed and tumor growth was examined. H&E-stained coronal brain sections show representative tumor xenografts from seven mice each group (left bottom panel). Tumor volumes were calculated (right panel). Data represent the means ± SD of seven mice. Immunoblotting analyses were performed with the indicated antibodies.

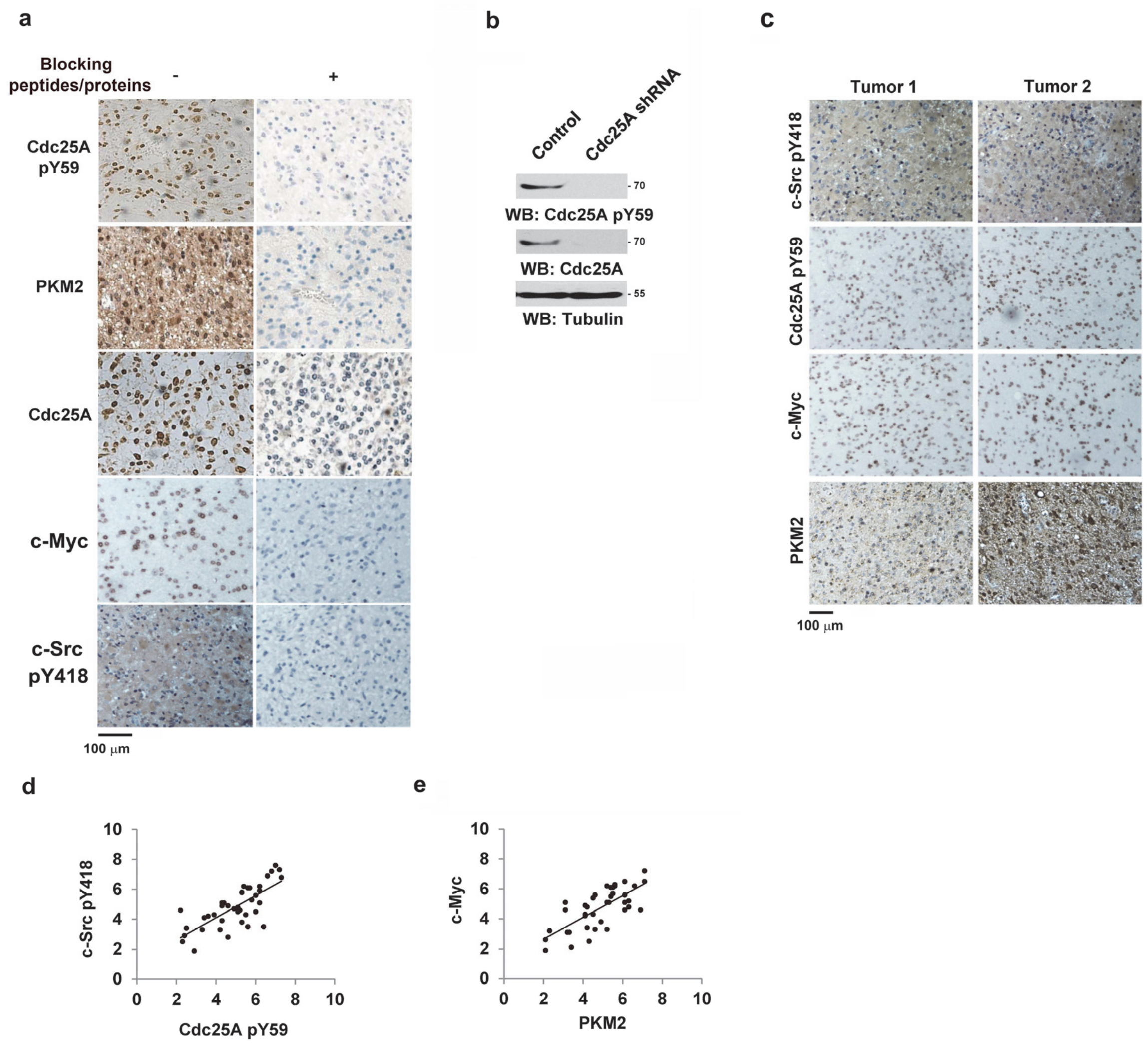

Supplementary Figure 9. Validation of antibody specificities and positively correlated expression of c-Src pY418, Cdc25A pY59, c-Myc, and PKM2.

(**a**) IHC analyses of human GBM tissues were performed with the indicated antibodies in the presence or absence of specific blocking peptides against PKM2, c-Myc, c-Src pY418 or Cdc25A pY59, or recombinant protein of Cdc25A.

(**b**) U87/EGFR cells with control shRNA or Cdc25A shRNA were treated with EGF for 4 h. Immunoblotting analyses were performed with the indicated antibodies.

(**c-e**) Immunohistochemical stainings of 40 GBM specimens with anti-phospho-c-Src Y418, anti-phospho-Cdc25A Y59, anti-PKM2, and anti-c-Myc antibodies were performed. (c), representative photographs of four GBM specimens; (d and e), semi-quantitative scoring (using a scale from 0 to 8) was carried out (Pearson product moment correlation test, d, $r = 0.75$, $P < 0.001$; e, $r = 0.73$, $P < 0.001$).

Figure 1A

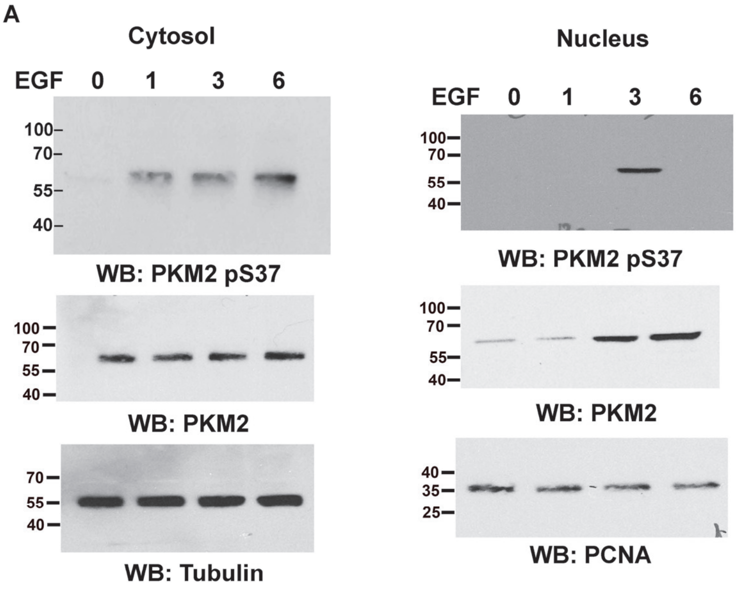

Figure 1C

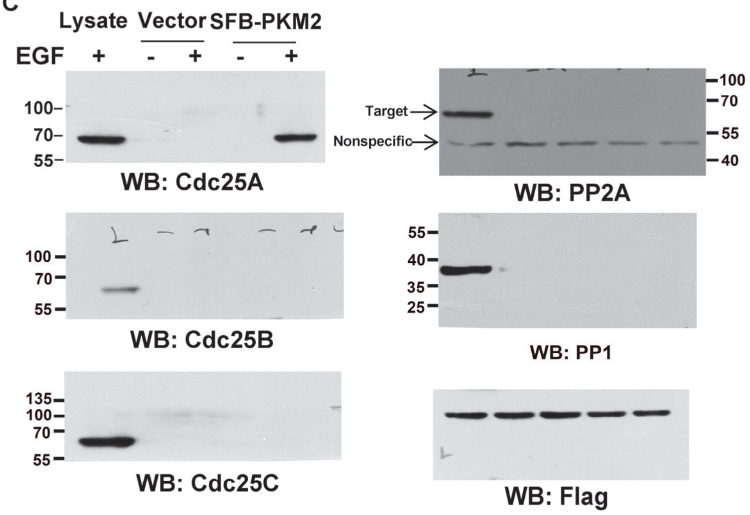

Figure 2H

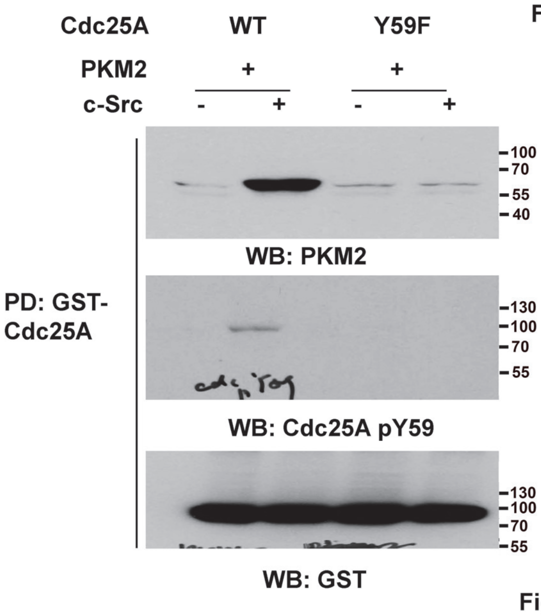

Figure 4A

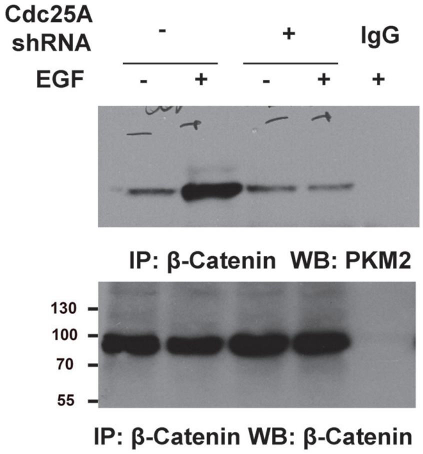

Figure 3D

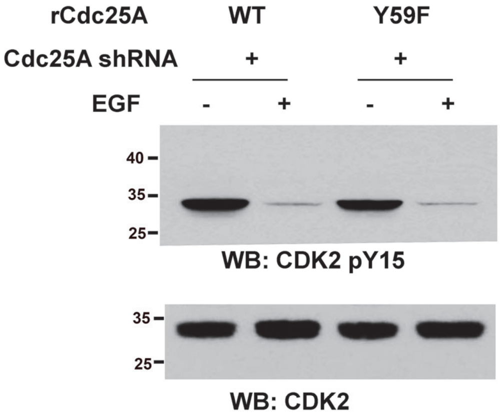

Figure 2K

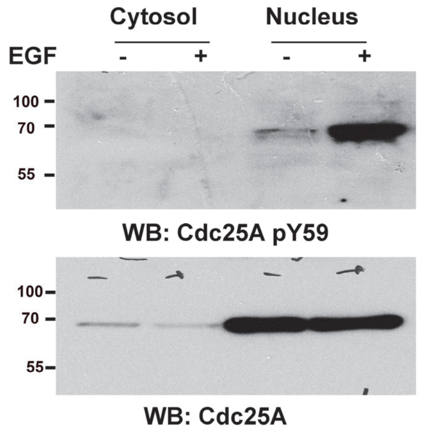

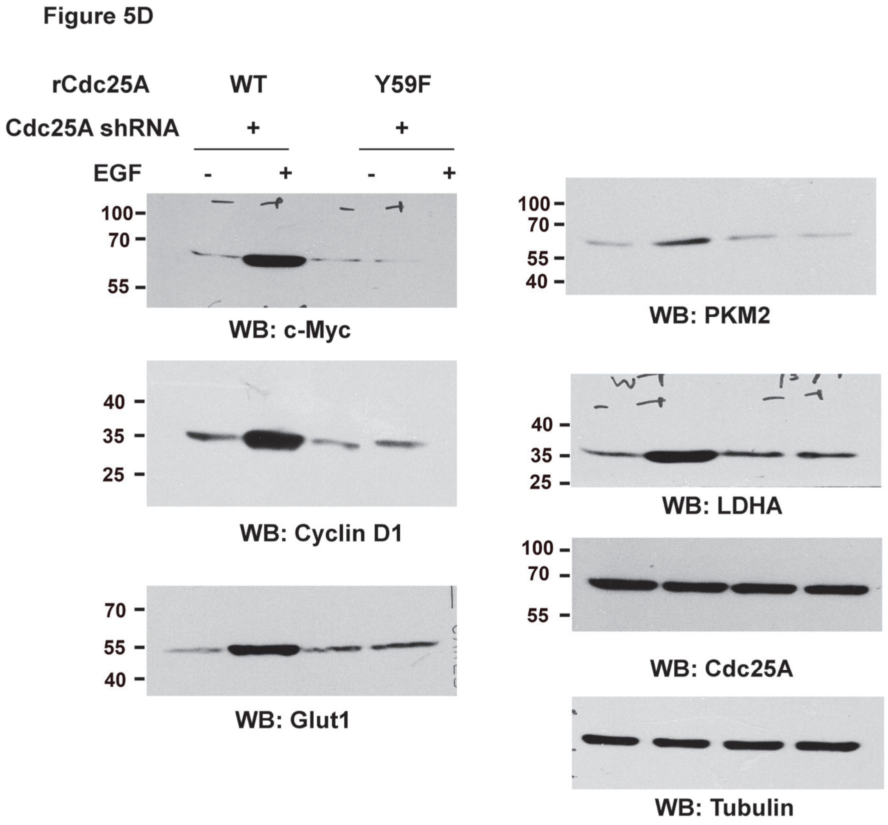

Supplementary Figure 10. Images of uncropped gels for key Western blots.
